# Single-cell transcriptomics analysis reveals that the tumor-infiltrating B cells determine the indolent fate of papillary thyroid carcinoma

**DOI:** 10.1186/s13046-025-03341-7

**Published:** 2025-03-11

**Authors:** Chunmei Li, Pei Wang, Zhizhong Dong, Weihan Cao, Yanjun Su, Jianming Zhang, Shuyan Zhao, Zhiyuan Wang, Zi Lei, Li Shi, Ruochuan Cheng, Wen Liu

**Affiliations:** 1 State Key Laboratory for Conservation and Utilization of Bio-resources and School of Life Sciences, Yunnan University, Kunming, Yunnan 650091 China; 2 Department of Radiation Oncology, Cancer Institute, The First Affiliated Hospital and College of Clinical Medicine of Henan University of Science and Technology, Luoyang, China; 3 Department of Thyroid Surgery, Clinical Research Center for Thyroid Diseases of Yunnan Province, The First Affiliated Hospital of Kunming Medical University, Kunming, Yunnan China; 4 Department of Ultrasound Imaging, The First Affiliated Hospital of Kunming Medical University, Kunming, Yunnan China; 5 Department of Pathology, The First Affiliated Hospital of Kunming Medical University, Kunming, Yunnan China; 6 Endocrine and Metabolic Diseases Clinical Medical Center of Yunnan, The First Affiliated Hospital of Kunming Medical University, Kunming, Yunnan China

**Keywords:** Papillary thyroid carcinoma, Indolent, Tumor-infiltrating B, Germinal center B

## Abstract

**Objective:**

Active surveillance (AS) offers a viable alternative to surgical intervention for the management of indolent papillary thyroid carcinoma (PTC), helping to minimize the incidence of unnecessary treatment. However, the broader adoption of AS is hindered by the need for more reliable diagnostic markers. This study aimed to identify the differences between indolent and progressive PTC and find new targets for biomarker development and therapeutic strategies.

**Methods:**

We used single-cell RNA sequencing (scRNA-seq) to analyze cellular differences in 10 early-stage PTC tumors. Findings were validated in an additional 25 tumors using cell co-culture, migration assays, immunofluorescence staining, flow cytometry, and analysis of data from The Cancer Genome Atlas (TCGA).

**Results:**

Tumor-infiltrating B cells (TIL-B), particularly germinal center B cells (GC-B), were more abundant in indolent PTC. These cells suppressed thyroid cell proliferation in both indolent and progressive cases, though indolent PTC had a higher capacity to recruit peripheral B cells. In indolent cases, TIL-B cells showed increased proliferation and formed clusters within tertiary lymphoid structures (TLS). PTPRC-CD22 interactions were identified as potential drivers of TIL-B cell proliferation. Markers linked to GC-B cells, such as *LMO2*, were highlighted as potential diagnostic and prognostic indicators for indolent PTC.

**Conclusion:**

This study provides insights into the cellular landscape of early-stage PTC, revealing distinct tumor and immune microenvironment features in indolent and progressive cases. These findings advance the understanding of indolent PTC biology and support the development of reliable diagnostic and prognostic biomarkers.

**Supplementary Information:**

The online version contains supplementary material available at 10.1186/s13046-025-03341-7.

## Introduction

In recent decades, there has been a marked increase in the incidence of papillary thyroid carcinoma (PTC) worldwide, while the mortality rate has remained essentially stable [1]. This increase is attributed mainly to the over-diagnosis of small, asymptomatic, low-risk PTCs, primarily due to the widespread use of high-resolution ultrasound imaging [2]. In China, up to 200,000 patients undergo unnecessary surgical procedures and long-term thyroid-stimulating hormone (TSH) suppression annually [3].

Several clinical trials have demonstrated that active surveillance (AS) can be a productive alternative to immediate surgery for asymptomatic patients with PTC [4–6]. These studies substantiate the hypothesis that a subset of PTCs demonstrate stable or slow growth and that only a minor proportion of these ultimately progress to the point where delayed surgery is necessary. Despite the benefits of AS in reducing overtreatment, the use of AS remains constrained globally due to the absence of diagnostic and prognostic clinical markers for indolent PTC.

The tumor volume doubling time (TVDT), as determined by computed tomography (CT), magnetic resonance imaging (MRI), or ultrasound, is a pivotal metric for differentiating between aggressive and slow-growing tumors [7, 8]. Recently, TVDT has been proposed as an indicator for determining the necessity for surgical intervention during AS of PTCs [9]. The calculation of TVDT during the initial 2- to 3-year period of AS can facilitate the determination of the optimal course of action, whether it be surgical intervention or modifications to the follow-up regimen indicated. Nevertheless, the necessity for a 2- to 3-year observation period precludes the possibility of completely alleviating the psychological and financial burden patients bear. It is of the utmost importance to determine whether patients present with indolent or progressive PTC at the time of diagnosis. Biological and molecular markers are more sensitive than imaging techniques and are commonly employed for early tumor screening [10]. However, no biological or molecular markers can differentiate between indolent and progressive PTCs at an early stage [11].

The PTC lesion is constituted by malignant cells and various immune cells, forming a complex ecosystem. The heterogeneity of tumor cells and the tumor microenvironment (TME) plays a critical role in shaping tumor behavior. In recent years, single-cell RNA sequencing (scRNA-seq) has emerged as a powerful tool for exploring the complex cellular landscape of PTC, uncovering distinct populations of malignant cells and immune cell types within the TME [12]. It has been demonstrated that tumor-infiltrating immune cells, including regulatory T cells (Tregs) and tumor-associated macrophages (TAMs), play a role in cancer cells' capacity to evade immune responses [13]. It is noteworthy that specific B cells in primary tumors and CD8^+^ T cells in both primary tumors and lymph nodes exhibit overexpression of inhibitory receptors [14]. Additionally, scRNA-seq has identified distinct developmental phenotypes of malignant thyrocytes, including a premalignant subtype [15]. Although specific markers for aggressive PTC, such as immune checkpoint molecules and critical transcription factors, have been identified, reliable biomarkers to differentiate between aggressive and indolent forms of PTC remain elusive [16–18].

In this study, we employed scRNA-seq on 10 primary tumor tissues of early-stage PTC to investigate the intertumoral heterogeneity of tumor cells and TME in indolent and progressive PTCs and to identify diagnostic markers for indolent PTC. The main cell types were meticulously delineated, and the indolent and progressive PTC cellular composition was subjected to rigorous analysis. The transcriptome features were also characterized, and communication between different cell types was explored. Moreover, the findings were validated in additional cohorts (25 primary tumors) through cell co-culture, cell migration assays, immunofluorescence (IF) staining, flow cytometry, and by analyzing data from The Cancer Genome Atlas (TCGA) database. Our data provides insights into the distinctive characteristics of early-stage PTC tumor cells and the TME, contributing to comprehending the biological basis of indolent PTC. Our findings will thus facilitate the diagnosis of indolent PTC.

## Results

### Tumor-infiltrating B cells enriched in indolent PTC

**Fig. 1:**
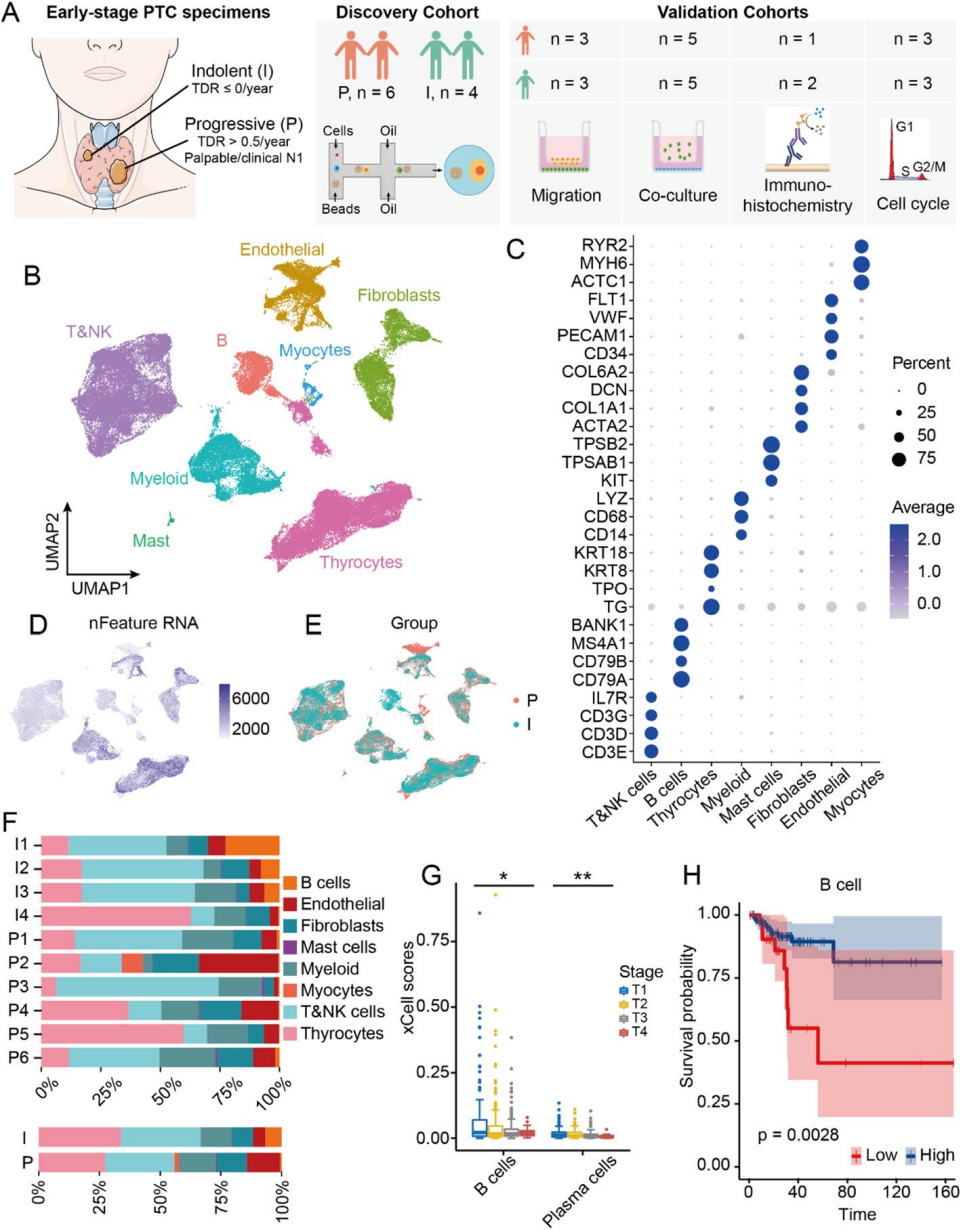
scRNA-seq profiling of the landscape of the early-stage PTCs. **A** Overview of the experimental design. **B** UMAP plot of the 74,714 cells from early-stage PTC primary tumor samples, 8 main cell types were identified and color-coded by cell type. **C** Dotplot of the marker genes expression for the identified cell types. The dot size and color represent the percentage of marker gene expression and the averaged scaled expression value, respectively. (D/E) UMAP view of all cells, (**D**) color-coded by number of genes detected in each cell. **E** color-coded by the patient group. **F** The distribution of cell composition for each patient sample (up) and the group (down). **G** Boxplot of the enrichment scores of B cells and plasma cells for different T-stages of PTCs. The PTC data from the TCGA database. Xcell was employed to analyze B cell and plasma cell enrichment. **H** Kaplan–Meier plot of DFS for PTC patients in the TCGA database. The DFS analysis was based on enrichment scores for all B cells (Methods). Cox proportional hazard models with a log-rank test were used for DFS analysis. PTC, Papillary Thyroid Carcinoma; TDR, Tumor Doubling Rate; DFS, Disease-Free Survival; I, Indolent; P, Progressive

To comprehensively investigate the heterogeneity of the ecosystem in indolent and progressive PTCs, we performed scRNA-seq (10 × Genomics, Methods, Supplementary Table S1) on 10 early-stage PTC specimens from 9 patients (Supplementary Table S2, discovery cohort, Fig. 1A, Methods), including 5 from our previous reported AS cohort [19] (Supplementary Fig. 1). Tumor progression was assessed using the tumor doubling rate (TDR), which is the inverse of TVDT and effectively quantifies alterations in tumor volume (Methods). 4 tumors were classified as indolent (TDR ≤ 0/year) and 2 as progressive (TDR > 0.5/year). One patient exhibited two distinct tumors, each with disparate clinical characteristics. One was classified as indolent, while the other was designated as progressive. Due to the limited number of progression cases in the AS cohort, 4 cases presenting as palpable tumors or clinical N1 disease were included as progressive samples. All the samples without autoimmune thyroiditis and the status of the tumor samples were reconfirmed through histopathological examination. The detailed clinical and pathological information, including tumor stage and tumor size, was provided in Table S2 (Supporting Information).

Following the quality control process, a total of 74,714 cells were subjected to analysis (Supplementary Table S1, Methods). Cell classification and marker gene identification were conducted using Seurat. The results were visualized using uniform manifold approximation and projection (UMAP). 8 main cell populations were identified according to the expression of canonical gene markers (Fig. 1B, C), including T/natural killer (NK) cells (*CD3D* ^+^, *CD3E* ^+^, *CD3G* ^+^, *IL7R* ^+^), thyrocytes (*TG* ^+^, *TPO* ^+^, *KRT8* ^+^, *KRT18* ^+^), myeloid cells (*CD14* ^+^, *CD68* ^+^, *LYZ* ^+^), B cells (*CD79A* ^+^, *CD79B* ^+^, *MS4A1* ^+^, *BANK1* ^+^), fibroblasts (*ACTA2* ^+^, *COL1A1* ^+^, *DCN* ^+^, *COL6A2* ^+^), endothelial cells (*CD34* ^+^, *PECAM1* ^+^, *VWF* ^+^, *FLT1* ^+^), myocytes (RYR2^+^, MYH6^+^, ACTC1^+^) and mast cells (*KIT* ^+^, *TPSAB1* ^+^, *TPSB2* ^+^). The gene expression profiles of immune cells and endothelial cells exhibited a lower degree of complexity in comparison to those of thyrocytes (Fig. 1D). Most cell populations were observed to be consistently present across different patients and between indolent and progressive PTC samples, although their relative proportions exhibited variation. It is noteworthy that myocytes were specifically identified in one sample (P2), which may indicate a potential sampling bias due to the inclusion of muscle tissue. Of particular interest was the observation that tumor-infiltrating B (TIL-B) cells were enriched in indolent PTC cases but nearly absent in progressive cases (Fig. 1E, F). Previous studies have documented the presence of B cells in PTC cases associated with Hashimoto's thyroiditis [20], indicating that TIL-B cells may play a unique role in the fate determination of PTC development.

We further analyzed bulk mRNA-seq data from the TCGA database and found that higher frequencies of B cells and plasma cells were associated with improved tumor staging in PTC patients (*P* = 0.013 and 0.002; Fig. 1G). Furthermore, survival analyses indicated that high enrichment of B cell, germinal center B (GC-B) cell and plasma cell gene sets were associated with improved disease-free survival (DFS) in PTC patients (HR = 3.59, 3.43, and 3.68; *P* = 0.003, 0.004, and 0.002; Fig. 1H, Supplementary Fig. 2). These findings suggest that TIL-B cells may be a pivotal component of the anti-tumor immune response in PTC.

### Heterogeneity of thyrocytes between indolent and progressive PTCs

**Fig. 2:**
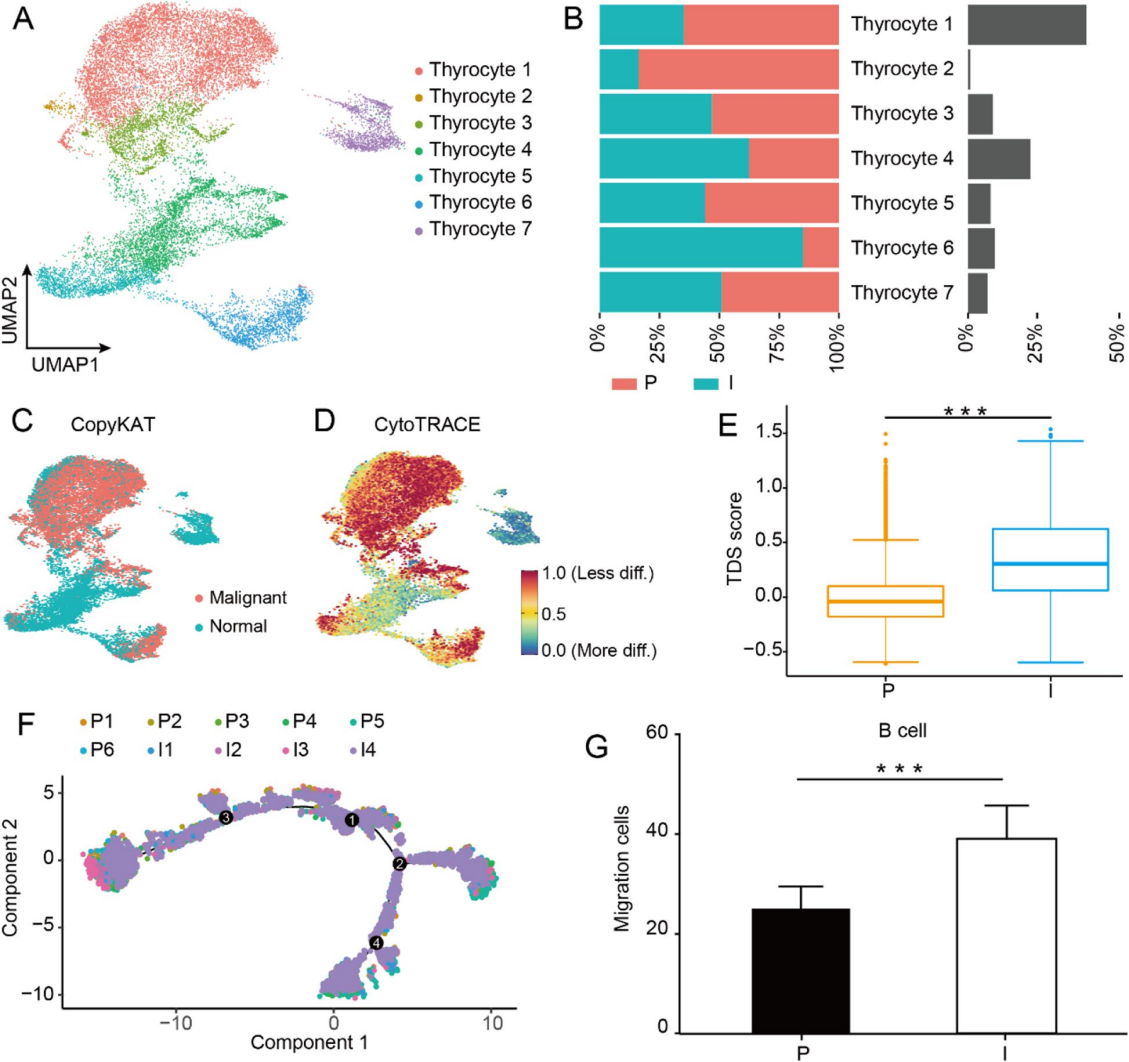
Thyrocytes heterogeneity between indolent and progressive PTCs. **A** UMAP plot of the total thyrocytes from early-stage PTC, color-coded by cell type. **B** The proportion of each thyrocyte cluster in the progressive (red) and indolent (cyan) group and the percentage of thyrocytes in total cells in each cluster (gray). **C**, **D** UMAP plot of all thyrocytes from early-stage PTC, (**C**) color-coded by inferred CNV, (**D**) color-coded by the predicted differentiation state via CytoTRACE. **E** Boxplots of TDS scores for thyrocytes of indolent and progressive PTCs. **F** The pseudo-time trajectories of all thyocytes. Thyrocytes are ordered along pseudotime trajectories, with the cells color-coded by patients. **G** Histogram of the number of migration peripheral blood B cells recruited by the primary tumor cells of indolent and progressive groups. A two-sided unpaired Wilcoxon test was performed to compare between groups. *** indicates *p*-value < 0.001. CNV, Copy Number Variation, TDS, Thyroid Differentiation Score.

Subsequently, we focused on the heterogeneity of thyrocytes between indolent and progressive PTCs. We further demarcated thyrocytes into 7 clusters based on the patterns of differentially expressed genes (DEGs) (Fig. 2A). GSVA Hallmark analysis revealed significant transcriptional differences across these clusters (Supplementary Fig. 3, Supplementary Table S3). For example, thyrocyte 2 showed increased activity in pathways associated with cell cycle regulation and DNA methylation. Thyrocyte 3 was marked by an abundance of pathways related to lymphocyte recruitment, including TNF signaling, which induces fibroblasts to produce lymphoid chemokines, and TGF-β signaling, which has been demonstrated to facilitate follicular helper T (Tfh) cell differentiation. Thyrocyte 4 was enriched in pathways associated with blood vessel formation, epithelial-mesenchymal transition (EMT), and immune inhibition. Thyrocyte 5 was mainly enriched in pathways related to cell proliferation and stress response. Notably, no apparent differences in cluster distribution were observed between the two groups (Fig. 2B).

To identify malignant and non-malignant thyrocytes, we used a series of integrated approaches. First, the copy number variants (CNVs) of the cells were compared using CopyKAT (Fig. 2C). Subsequently, CytoTRACE and Thyroid Differentiation Score (TDS) were employed to predict the differentiation status of the thyrocytes, and the transcriptome profile of the cell clusters was examined. Finally, four malignant thyrocyte clusters (Thyrocyte 1, 2, 3, and 6) were identified. The poorly differentiated cells were concentrated in the malignant clusters (Fig. 2D), which is in accordance with previous findings [15, 21]. The indolent group exhibited significantly higher TDS scores than the progressive group, indicating more significant differentiation (Fig. 2E). The proportion of malignant thyrocytes was found to be comparable between the indolent and progressive groups (Fig. 2B). Furthermore, BRAF^V600E^ and RAS mutation profiles were examined [22]. The results showed a lower prevalence of BRAF^V600E^ and a higher incidence of RAS mutations in the indolent group compared to the progressive group (Supplementary Fig. 4).

Given the potential of dedifferentiation in PTC, which may drive the transformation of indolent tumors into progressive forms [23], we employed the Monocle algorithm to elucidate the differentiation trajectory of all thyrocytes and further investigate their differentiation potential. The results demonstrated that four distinct molecular states were identified in two groups (Supplementary Fig. 5). No pivotal molecular events determining cell fate were observed. Similar cell trajectories were identified in both groups (Fig. 2F). As illustrated in the UMAP plot and cell trajectories, the benign cluster thyrocytes 5 and thyrocytes 4 exhibited characteristics associated with tumor initiation. Furthermore, the gene signatures of the typical thyroid epithelial markers *TG*, *TSHR*, and *IYD* and the PTC differentiation-related *TFF3* and *FHL1* demonstrated a downward trend from thyrocytes 4 and 5 to thyrocytes 3 (Supplementary Fig. 6). This dedifferentiation process was similar to that previously reported by Pu W et al*.* on the developmental process of PTC [15].

### Thyrocytes exhibited augmented capacity for the recruitment of peripheral B cells in indolent PTC

Given the notable discrepancies observed in TIL-B cells between the indolent and progressive groups, we postulated that thyrocytes may demonstrate disparate capacities to recruit peripheral B cells between the two groups. To further validate this hypothesis, we collected 3 fresh PTC tissue samples from each group (Supplementary Table S2 validation cohort 1) and isolated primary thyroid cells. B cells from the peripheral blood of the same healthy volunteer were co-cultured with primary thyroid cells from both groups in a 1:1 ratio for the trans-well migration assay (Methods). The results demonstrated that thyrocytes from indolent tumors exhibited a significantly enhanced capacity to recruit peripheral B cells compared to those from progressive tumors (Fig. 2G). However, an analysis of B cell recruitment-related receptors (*CXCR4*, *CXCR5*, *CCR6*, and *CCR7*) in our scRNA-seq data revealed no significant differences between the two groups (Supplementary Fig. 7). This indicates that the increased recruitment of peripheral B cells observed in indolent tumors may be exclusively attributable to the thyroid cells.

### TIL-B cells suppress thyrocyte proliferation

**Fig. 3:**
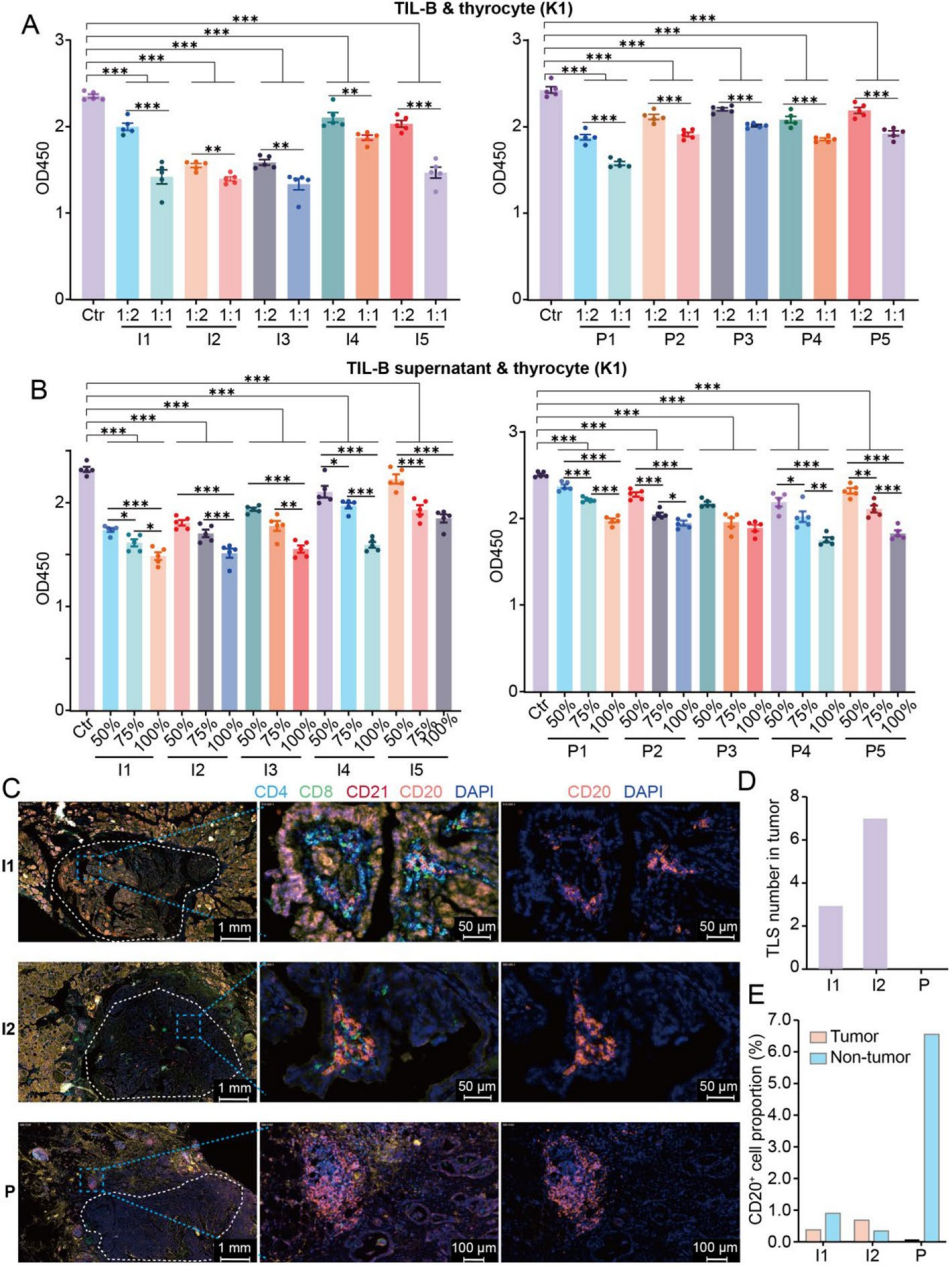
TIL-B cells suppress thyrocyte proliferation in both groups but cluster to format the TLSs in indolent PTC. **A**, **B** Histogram of the OD450 absorbance in K1 cells that co-cultured with different proportions of TIL-B cells (**A**) or TIL-B cells culture supernatants (**B**) from indolent (left) and progressive (right) PTCs. Cell growth of K1 cells was inhibited by TIL-B cells and culture supernatants from both two groups. The cell growth of K1 cells was determined by CCK8 assays. **C** Multiplex immunofluorescence analysis of the location of B cells and T cells in the tumor (inside the white dotted circle) and adjacent tumor tissues from indolent (I1, I2) and progressive (P) PTCs. Antibodies against CD4, CD8, CD20, and CD21 were used; CD4 and CD8 were marked for T cells, and CD20 and CD21 were marked for B cells. Cell nuclei counterstained with DAPI. TLS, characterized by B cell aggregated and surrounded by T cells, was exclusively present in the tumor region of indolent PTCs. **D** Histogram of the number of TLSs in the tumor areas of indolent and progressive PTCs. **E** Histogram of the proportion of CD20^+^ B cells in tumor region of indolent and progressive PTCs. A two-sided unpaired Wilcoxon test was performed to compare between groups. * indicates *p*-value < 0.05, ** indicates *p*-value < 0.01, *** indicates *p*-value < 0.001. TLSs, tertiary lymphoid structures. Ctr, negative control

To ascertain the direct effect of TIL-B cells on thyrocytes, we isolated TIL-B cells from 10 fresh PTC tissues (5 indolent and 5 progressive, Supplementary Table S2 validation cohort 2). Subsequently, the TIL-B cells were co-cultured with the PTC cell lines (K1 and B-CPAP) in vitro, and the proliferative activity of PTC cells was quantified using CCK-8 assays (Methods). The results demonstrated that TIL-B cells from both indolent and progressive PTCs inhibited the growth of K1 and B-CPAP cells in a concentration-dependent manner (Fig. 3A, Supplementary S8). In addition, supernatants from the TIL-B cells of both PTC types were collected and co-cultured with the same cell lines. These supernatants similarly inhibited cell proliferation in a concentration-dependent manner. (Fig. 3B, Supplementary S8). This finding suggests that TIL-B cells suppress thyroid cell proliferation through direct cell–cell interaction and secretion of inhibitory proteins. Overall, these findings indicate that TIL-B cells from both indolent and progressive PTCs possess anti-tumor properties. The clinical presentation of PTC as indolent may mainly depend on the quantity of TIL-B cells in the TME.

### Increased formation of tertiary lymphoid structures in indolent PTC

Recent studies have demonstrated a correlation between the presence of tertiary lymphoid structures (TLS) in tumors and an improved prognosis in patients with solid cancers [24–26]. Nevertheless, the function of TLS in PTC remains insufficiently investigated. To investigate this, we calculated TLS scores for PTCs using TCGA data and found that high TLS gene signature enrichment correlated with improved DFS in PTC patients aged ≥ 55 years (HR: 3.983, *P* = 0.0016, Supplementary Fig. 9).

To gain further insight into the spatial distribution of TIL-B cells and other immune components, we performed multiplex immunofluorescence staining on both indolent and progressive PTC samples (2 indolent and 1 progressive, Supplementary Table S2 validation cohort 3, Methods). In indolent PTC, distinct lymphocyte aggregation areas were observed, characterized by clusters of B cells surrounded by T cells, which are vital indicators of TLS formation (Fig. 3C, D). In contrast, in progressive PTC, B cell aggregation was observed exclusively at the adjacent tissues of the tumor, with no evidence of TLS formation within the tumor (Fig. 3C, D). The number of B cells in the tumor was consistent with the results of our scRNA-seq, which showed a significant absence of TIL-B cells in the tumor region of progressive PTC (Fig. 3E).

### TIL-B cells showed higher proliferative capacity in indolent PTC

**Fig. 4:**
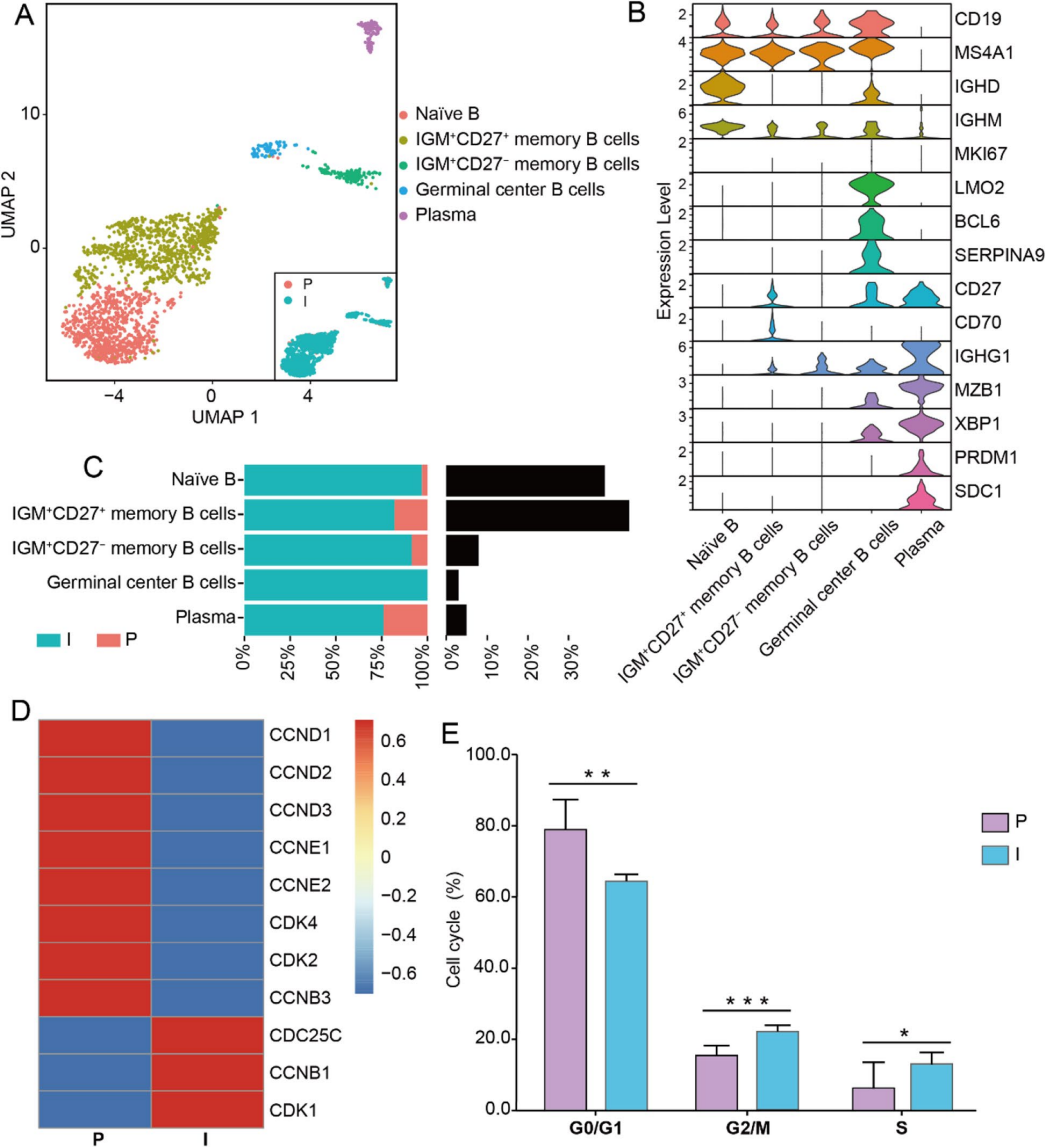
Heterogeneity of TIL-B cells between indolent and progressive PTCs. **A** UMAP plot of the TIL-B cells from early-stage PTCs, color-coded by cell type and groups. **B** Violin plots of the representative marker gene expression levels across the clusters of TIL-B cells. **C** The proportion of each TIL-B cluster in the indolent (cyan) and progressive (red) group, and the number of TIL-B cells in total cells in each cluster (gray). **D** Heatmap of cell cycle-related gene expression in TIL-B cells from indolent and progressive PTCs. **E** Histograms of the cell cycle distribution of all B cells from the progressive group and indolent group. A two-sided unpaired Wilcoxon test was performed to compare between groups. * indicates *p*-value < 0.05, ** indicates *p*-value < 0.01, *** indicates *p*-value < 0.001. TIL-B cells, tumor-infiltrating B cells

We further performed unsupervised clustering and subdivided B cells into 5 subgroups based on the patterns of DEGs (Fig. 4A, B, Supplementary Table S4, Supplementary Fig. 10). It is noteworthy that all B cell clusters were concentrated in the indolent PTC group, with the GC-B cell cluster exclusively present in this group (Fig. 4A, C). Gene set enrichment analysis (GSEA) revealed that GC-B cells exhibited a preferential upregulation of pathways associated with the cell cycle and mitotic processes, including E2F_TARGETS, G2M_CHECKPOINT, and MITOTIC_SPINDLE (Supplementary Fig. 11, Supplementary Table S5). Given the pivotal role of GC-B cells in mediating changes in the TME and promoting TLS formation [24], we postulated that they may influence the status of all TIL-B cells. To further validate this hypothesis, we analyzed the expression of cell cycle-related genes in all TIL-B cells. In the progressive group, genes associated with the G1 phase, such as D-type cyclins (*CCND1, CCND2,* and *CCND3*), exhibited high expression levels. In contrast, the indolent group exhibited elevated expressions of genes associated with the G2/M phase, including *CCNB1*, *CDK1*, and *CDC25C* (Fig. 4D). This indicates that TIL-B cells may demonstrate disparate proliferative capabilities in indolent and progressive PTCs.

Furthermore, we isolated TIL-B cells from 6 fresh PTC tissues (3 progressive and 3 indolent, Supplementary Table S2 validation cohort 4) and analyzed their cell cycle distribution by flow cytometry (Methods). The results demonstrated a heightened proportion of TIL-B cells in the G0/G1 phase and a diminished proportion in the G2/M phase in the progressive group compared to the indolent group (Fig. 4E, Supplementary Fig. 12). This indicates that the proliferation of TIL-B cells in progressive PTCs was arrested in the G1 phase, with reduced mitotic activity. In conclusion, the results suggest that TIL-B cells from indolent PTC exhibit a higher proliferative capacity, which the presence of GC-B cells may influence.

## PTPRC-CD22 trans-binding might be the initiate factor of TIL-B cell proliferation

**Fig. 5:**
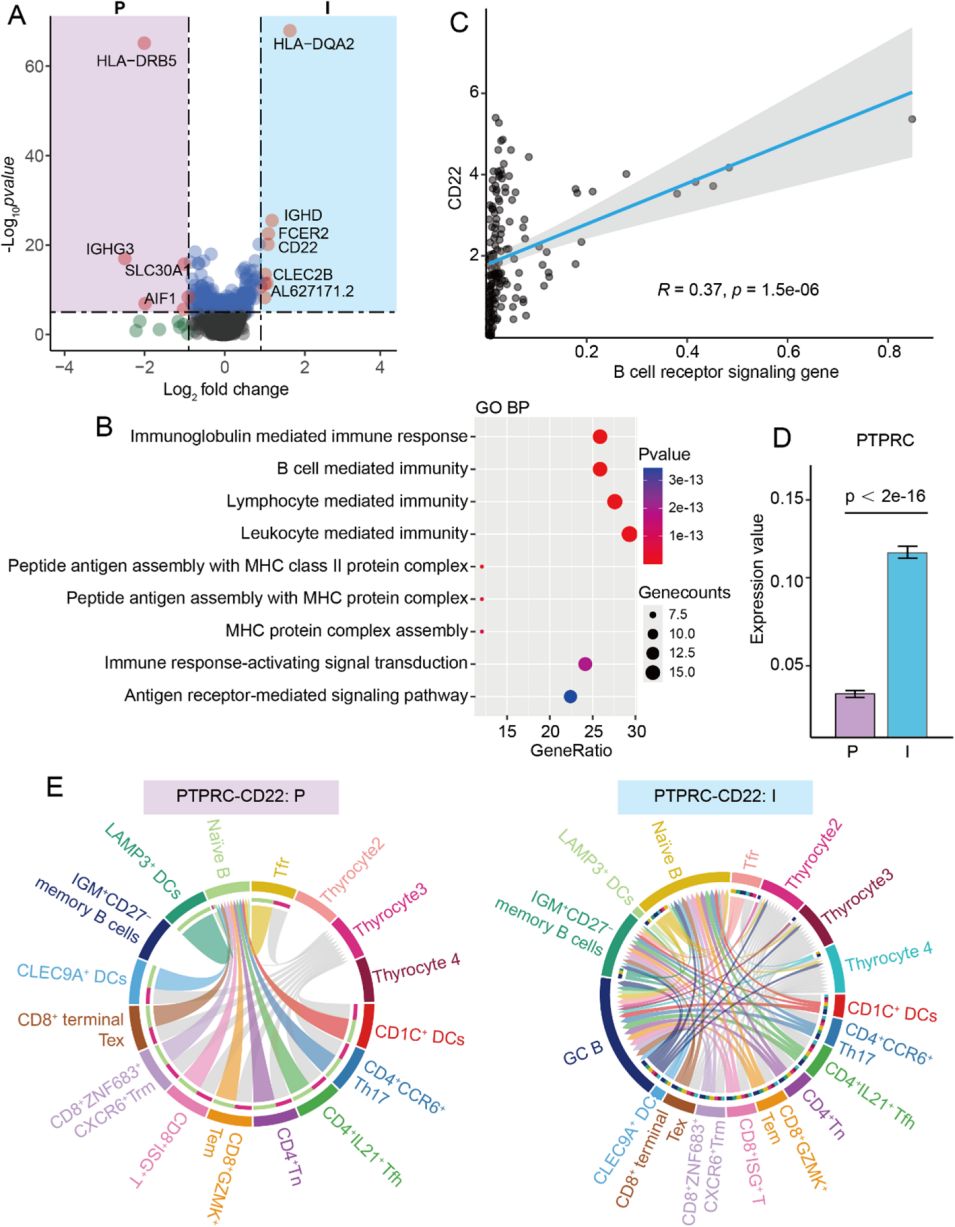
PTPRC-CD22 trans-binding in indolent and progressive PTCs. **A** The Volcano plot of the DEGs of TIL-B cells between indolent and progressive PTCs, red dots representing the most up-regulated genes in the two groups. **B** Bubble plots of GO results of up-regulated genes in TIL-B cells of the indolent PTC. **C** The scatter plot showed a positive correlation between CD22 expression and the B cell receptor signaling gene for PTC patients of the TCGA database. **D** Histograms of PTPRC expression in thyrocytes of the progressive and indolent groups. A two-sided unpaired Wilcoxon test was performed to compare between groups. **E** Chord diagram of the possible PTPRC-CD22 trans-binding interaction among cells in progressive (P) and indolent (I) PTCs. Interactions with B cells were colored, and others were gray. DEGs, Differentially Expressed Genes.

Subsequently, we investigated the transcriptional differences in TIL-B cells between indolent and progressive PTCs. The most significantly up-regulated genes in indolent tumors were *HLA-DQA2*, *IGHD*, *FCER2*, *CD22*, and *CLEC2B*. In contrast, the progressive tumors exhibited the most significant increase in expression of *HLA-DRB5*, *IGHG3*, *SLC30A1*, and *AIF1* (Fig. 5A, Supplementary Table S6). Among these genes, *CD22*, exclusively expressed in B cells, functions as an inhibitory molecule in B cell receptor signaling. It is commonly the target of treatments for conditions characterized by B cell hyperproliferation, including B cell malignancies, organ transplants, and autoimmune diseases [27, 28]. However, Gene Ontology (GO) analysis indicated that the up-regulated genes in indolent TIL-B cells were linked to B cell receptor signaling (Fig. 5B). Additionally, Pearson correlation analysis of data from the TCGA database indicated a positive correlation between CD22 expressions and the enrichment of B cell receptor signaling gene in PTC (Fig. 5C). CD22 can bind to sialic acid ligands on the surface of the same or other cells, a process known as cis-binding and trans-binding, respectively. Cis-binding generally inhibits B cell receptor signaling, whereas trans-binding promotes B cell proliferation [29, 30]. It is noteworthy that PTPRC (known as CD45), the ligand for CD22, was significantly up-regulated in indolent PTC (Fig. 5D), indicating the potential for PTPRC-CD22 trans-binding interactions in indolent TIL-B cells.

To gain further insight into the distinctions in PTPRC-CD22 trans-binding between the two groups, we utilized the CellPhoneDB tool to examine cell-to-cell communication. First, we conducted a clustering analysis to identify finer subtypes of T/NK cells, myeloid cells, fibroblasts, and endothelial cells (Supplementary Fig. 13). Subsequently, we analyzed the intercellular communication networks of all cell subsets with respect to all ligand-receptor pairs. The results showed that among the most significantly up-regulated genes, only CD22, acting as a ligand (CD22-PTPRC), mediated the remarkable differences in cell communication between indolent and progressive PTCs (Supplementary Fig. 14-S17). Overall, CD22-PTPRC trans-binding interactions were more frequent in indolent PTC than in progressive PTC. In indolent PTC, the CD22-PTPRC trans-binding interactions were observed between most B subsets (naïve B, IGM^+^ CD27^−^ memory B, and GC-B) and other cell types, including T cell subsets, DC subsets, and thyrocytes (thyrocyte 2, 3, and 4). In contrast, in progressive PTC, the interactions were restricted to those between naïve B cells and a smaller number of other cell types, with only thyrocyte 3 being involved in thyrocyte interactions (Fig. 5E). This finding suggests that the trans-binding of CD22 with exogenous PTPRC in indolent PTC may play a pivotal role in initiating TIL-B cell proliferation.

### GC-B cell signatures are associated with improved DFS in patients with PTC

**Fig. 6:**
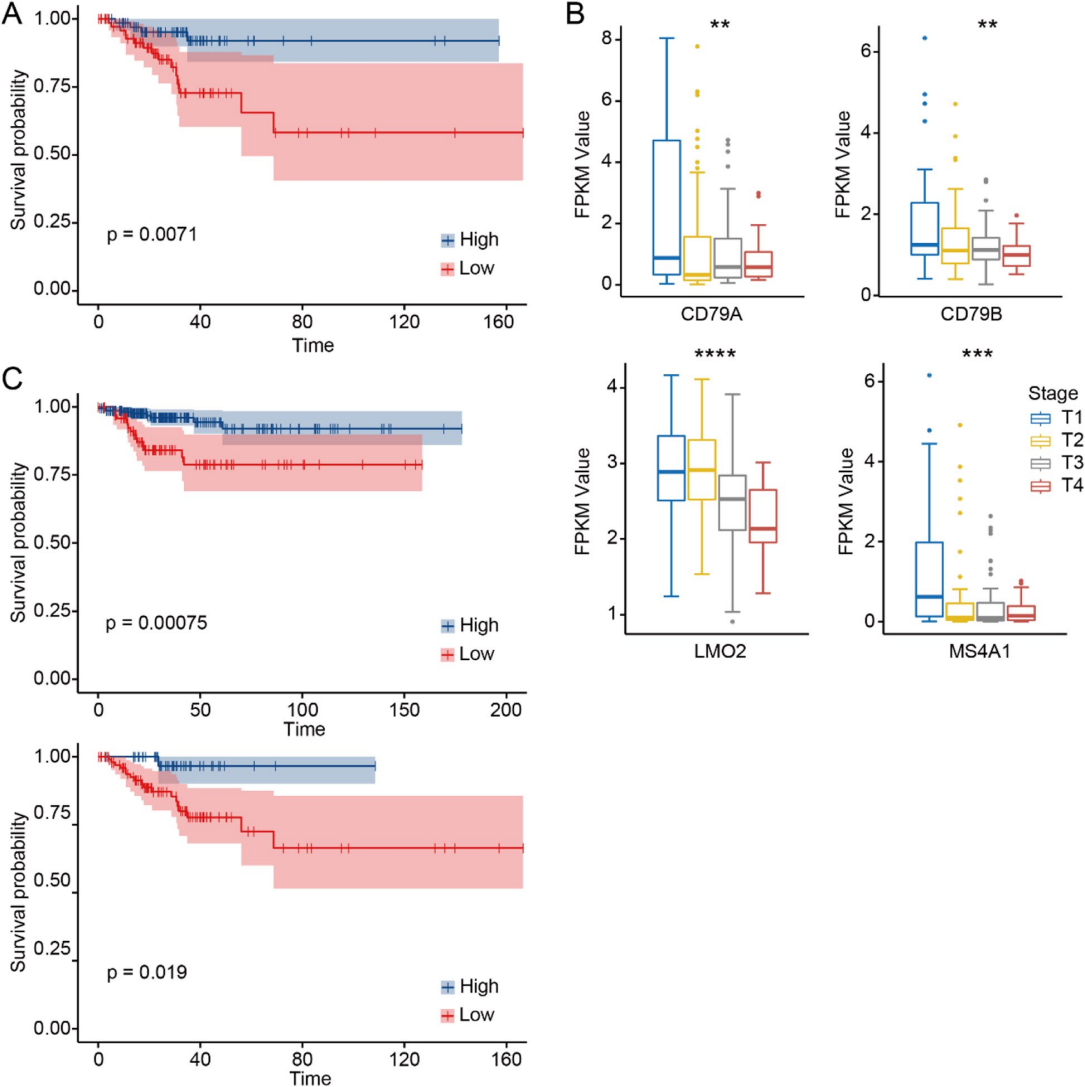
GC-B cell enrichment positively associated with improved T-stage and DFS in patients with PTC. Kaplan–Meier plot of DFS (**A**) for PTC patients in the TCGA database based on enrichment scores for GC-B cells using the Xcell method. Gene sets used to calculate enrichment scores were derived from our scRNA-seq analysis and applied to bulk mRNA-seq data from the TCGA database (Methods). **B** Boxplots of the GC-B-specific gene expression in different T-stages of PTCs. The PTC data from the TCGA database. The results showed that GC-B cells are positively associated with improved T stages for the PTC patients. A two-sided unpaired Wilcoxon test was performed to compare between groups. ** indicates *p*-value < 0.01, *** indicates *p*-value < 0.001. **C** Kaplan–Meier plot for DFS of PTC patients with high expression of *LMO2 *aged <55 and ≥55 in the TCGA database. Cox proportional hazard models with a log-rank test were used for DFS analysis

Given the specific presence of GC-B cells in the indolent PTC, this suggests that GC-B cells may play a key role in determining the indolent fate of PTC, with potential clinical relevance. We selected 21 GC-B cell-specific genes (Supplementary Table S4) and calculated enrichment scores (as GC-B enrichment scores) for each bulk mRNA sample from the TCGA database. A high GC-B enrichment score was associated with longer DFS in PTC patients (HR: 4.016, *P* = 0.0071, Fig. 6A). In addition, the high frequencies of 9 of the above 21 GC-B-cell-specific genes, including *BASP1*, *CD79A*, *CD79B*, *LMO2*, *LRMP*, *MS4A1*, *RGS13*, *TCL1A*, and *VPREB3*, were significantly associated with the improved T-staging of PTC (Fig. 6B, Supplementary Fig. 18). Among the top 9 GC-B cell genes, high expression of *LMO2* was particularly associated with improved DFS in PTC patients aged < 55 and ≥ 55 (HR: 3.751, *P* = 0.00075 and HR:7.543, *P* = 0.019, respectively; Fig. 6C), suggesting its potential as a diagnosis and prognostic marker for indolent PTC.

## Discussion

The advent of single-cell sequencing technology has illuminated the complex cellular landscape of thyroid cancers, revealing new layers of heterogeneity and potential drivers of recurrence and changes in the TMEs [12, 14, 15]. However, approximately 86.7% of female PTC cases in China and 76.0% in the United States are estimated to be overdiagnosed, particularly in low-risk instances [2]. Most low-risk PTCs demonstrate low malignant potential, excellent prognosis, and a disease-specific mortality rate of less than 1% [31]. Thus, distinguishing truly indolent PTC is essential for appropriate clinical management. In this study, we conducted an in-depth single-cell transcriptomic analysis to compare the tumor ecosystems of early-stage indolent and progressive PTCs. We found that the TME, especially immune cell activity, plays a central role in PTC behavior.

Several previous studies have reported that the enrichment of TIL-B cells in PTC is negatively correlated with lymph node metastasis and positively associated with improved survival prognosis. However, studies on the relationship between TIL-B cells and tumor size or invasiveness have yielded conflicting results, making the value of TIL-B cells as a predictive marker for PTC uncertain [32–34]. These differences in findings can be attributed to factors such as sample storage time, variations in cell aggregation classification, and the inherent uncertainties of single-study methodologies. In our study, we conducted a comprehensive analysis using fresh early-stage PTC tumor tissues, including single-cell sequencing and various cell experiments. We validated the results through the TCGA database. Our work confirmed the crucial role of TIL-B cells, particularly GC-B cells, in the indolent behavior of PTC. Elevated TIL-B cells were observed clustering into TLSs in indolent PTCs, where they seemed to inhibit thyrocyte proliferation, suggesting an immune equilibrium that may help prevent tumor progression [35]. B-cell responses are characterized by two distinct pathways: GC-dependent and GC-independence. The former is responsible for the production of antigen-specific plasma and memory B cells [36–38]. The involvement of GC-B cells indicates the presence of an antigen-specific immune response, which may contribute to maintaining immune stability in indolent PTC [39, 40]. This equilibrium may serve as a mechanism to slow disease progression. Further research into the antigen-specific response could lead to the development of novel therapeutic approaches for the treatment of PTC. Although TIL-B cells, and by extension TLS, appear to be critical to immune equilibrium, our findings indicate that other immune cells also play essential roles, which warrant further investigation.

A notable diversity was observed among the thyrocytes. Our study found a significant difference in the ability of thyrocytes to recruit B cells between indolent and progressive PTC. The mechanisms regulating T and innate immune cell migration are well established; however, those governing B cell migration remain poorly understood [41]. Transcriptomic analysis showed no significant variation in chemokine receptor expression among TIL-B cells between indolent and progressive cases, indicating that differences in TIL-B cell abundance are attributable to variations in thyroid cell populations. It may be advantageous to enhance the B-cell recruiting potential of thyroid cells as a strategy for PTC treatment.

Our cell communication analysis highlighted unique interactions between TIL-B cells and specific thyrocyte populations in indolent PTC. Thyrocytes 2, 3, and 4 frequently interacted with TIL-B cells through PTPRC-CD22 trans-binding in indolent PTC, while only thyrocyte 3 showed this interaction in progressive PTC. This mechanism, which promotes B cell proliferation, may contribute to the unique immune environment in indolent PTC. Specifically, thyrocyte 3 was associated with lymphocyte recruitment, thyrocyte 2 exhibited activity linked to cell proliferation and DNA methylation, and thyrocyte 4 displayed premalignant traits [15]. These differences in thyrocyte populations could influence TIL-B cell abundance and ultimately define the sluggish behavior of the tumor. Further research is necessary to clarify these differences and explore their implications for PTC progression and therapeutic options.

Our findings suggest that GC-B cells may serve as an effective diagnostic marker for identifying patients with low-risk, indolent PTC. Several GC-B-specific genes show potential as diagnostic markers, particularly the *LMO2*, which showed remarkable predictive efficacy (HR 7.543, *P* = 0.019 for DFS) in the ≥ 55 years age group that usually has a high incidence of adverse events in PTC. These markers, if further validated, could contribute to more sophisticated patient management. In the preoperative, this approach could guide treatment planning, allowing some patients to avoid surgery in favor of conservative management. However, the low abundance of GC-B cells in tumors and the limitations of fine-needle aspiration biopsy (FNAB) pose challenges for using GC-B cells as a reliable diagnostic tool. Future research should focus on developing more sensitive methods to detect GC-B cells, including exploring GC-B cells in the peripheral blood for non-invasive detection. Additionally, intraoperative identification of GC-B cells might aid surgical decision-making, potentially allowing for thyroid preservation in select PTC patients. For instance, rapid immunohistochemistry could detect GC-B cell markers after initial tumor removal, helping surgeons decide on the extent of tissue resection [42]. Postoperatively, GC-B cell markers could help assess recurrence risk, aiding in risk stratification and potentially avoiding unnecessary endocrine suppression in indolent PTC cases. This approach would enhance patient outcomes and minimize treatment side effects, promoting a more personalized treatment strategy.

It is important to acknowledge the limitations of our study. First, the sample size was limited, as only a small number of patients in our prospective AS cohort underwent surgery, making it difficult to obtain indolent PTC samples. Sampling bias may also affect the generalizability of our findings, especially if adjacent tissues were unintentionally included. Additionally, the absence of longitudinal tumor samples restricts our ability to track TIL-B cell diversity in the early stages, limiting the construction of a comprehensive immunological profile of various tumor stages. Moreover, the necessity for further validation over extended periods cannot be overstated to guarantee the accuracy and reliability of the findings. Although we compared our findings with data from TCGA to establish clinical relevance, the absence of detailed clinical information, such as the intervals between tumor observations before surgery, limits the precision of our evaluation of indolent PTC.

In conclusion, our study provides a comprehensive overview of the tumor microenvironment in indolent PTC. It emphasizes the pivotal role of TIL-B cells in defining the indolent nature of these tumors. These findings offer new insights into the heterogeneity of PTC and represent a significant advance in our understanding of the "mystery of indolence" in PTC. We hope our research will contribute to developing precision diagnostic tools and therapies for PTC, improving patient outcomes.

## Methods

### Human tumor specimens

As previously outlined, we conducted a prospective observation cohort with AS management for patients with low-risk PTC [19]. Patients underwent ultrasound monitoring at 6- to 12-month intervals. In this AS cohort, patients who underwent thyroid surgery due to changes in willingness to monitor (State 1) or disease progression (State 2) were recruited from September 2020 to June 2023 at the First Affiliated Hospital of Kunming Medical University (Fig. 1).

The status of the tumor was evaluated as either indolent or progressive by calculating the tumor volume doubling rate (TDR), which is the inverse of the TVDT (http://www.kuma-h.or.jp/English). In State 1, the tumor is indolent, TDR ≤ 0/year; In State 2, the cancer is rapidly enlarging, TDR > 0.5/year. Tumor samples with State 1 or State 2 were collected from the prospective AS cohort in our center, and all patients underwent at least three ultrasound evaluations. Considering that a limited number of progressing events were observed in the AS cohort, additional palpable or clinical N1 cases were obtained as non-indolent samples (State 3, clinical disease) during the same period. The status of the tumor samples was further confirmed through histopathological tests, with the results independently verified by two experienced pathologists.

In this study, the samples were divided into two groups following the clinical practice guidelines for patients with PTC, which were deemed appropriate for AS (State 1, indolent group) or immediate surgery (State 2&3, progressive group) management.

In the sequencing cohort, 10 fresh surgical specimens (primary tumors) from 9 patients qualified for quality control were sequenced and incorporated in further analyses (Supplementary Table S1). The indolent group comprises 4 T1-stage cases that exhibited no evidence of tumor progression (stable or shrinking) over the observation period (24–71 months). The progressive group comprises 2 T1-stage cases with tumor progression during the observation period (progressing) and 4 palpable, symptomatic T2 or N1b-stage cases (progressed).

An additional 25 fresh samples were utilized for cell experiments and multiple immunofluorescences. The clinical information, including demographics, medical history, tumor clinicopathologic characteristics and observation time, ultrasound, and laboratory data, was summarized in Supplementary Table 2.

### Sample preparation

Fresh samples were washed twice with 10% fetal bovine serum (FBS) in Dulbecco's Modified Eagle Medium (DMEM, Gibco). Each sample was then cut and subjected to enzymatic digestion with a 10 mL digestion medium containing 1 mg/mL collagenase and 2 mg/mL Dispase II (sigma). Subsequently, the samples were incubated at 37 °C with intermittent pipetting for 15-min intervals until the tissue pieces had disappeared. The suspended cells were filtered through a 40 µm cell-strainer nylon mesh (BD) and centrifuged at 500 g for 5 min. After removing the supernatant, the cell pellet was washed twice with 1 × PBS containing 0.4% BSA (Gibco).

### Libraries preparation, reads mapping, quality control, and clustering

The cell suspension of each sample was subjected to the Gel Bead Kit V3 (10 × Genomics) for library preparation according to the standard protocols. The single-cell libraries were sequenced on Illumina NovaSeq 6000 Systems using paired-end sequencing (150nt). The gene-barcode matrices were generated by the Cell Ranger toolkit (v3.1), which aligned the droplet-based sequencing data against the GRCh38 human reference genome and counted the unique molecular identifiers (UMIs) for each cell. The Seurat R package (v4.0.0) was used for quality control procedures and downstream bioinformatic analyses. Specifically, cells with less than 500 unique molecular identifiers (UMI) counts, or over 10% mitochondrial UMIs, were considered low-quality cells and removed. The DoubletFinder (v2.0) package [43] was utilized to remove the potential doublets with the default settings. After these quality control procedures, we employed a normalization method, "SCTransform," that normalized the feature expression for each cell by the total expression. The RunPCA function was applied to reduce the dimensionality of the datasets. Subsequently, the batch effect of the data was removed using the "Harmony" package. Finally, the data of the first 30 dimensions were utilized for unsupervised Louvain clustering and visualized using uniform manifold approximation projection (UMAP) with default parameters. We identified the major cell types in Fig. 1 according to classical marker genes.

### Thyrocyte analyses

The genome-wide copy number profiles are computed from the gene expression UMI matrix using the Bayesian segmentation approach, CopyKat V0.1.0 [44]. CytoTRACE was used to estimate the transcriptional diversity of each cell in terms of differential or stemness status [45]. The cells were given a CytoTRACE score according to their differentiation potential. Gene signatures of single cells were quantified to score the hallmark gene set by applying the single-sample gene set variation analysis (ssGSVA), which calculated the signature enrichment scores of individual single cells independently without normalization across cells. The AddModule Score function of the Seurat package was employed for TDS, which uses the expression profiles of 16 thyroid function-related genes to quantify relationships between thyroid differentiation and diverse genetic events [46]. The Monocle2 package (v2.14.0) performed the trajectory analysis to reveal the cell-state transitions [47]. All software runs according to official instructions with default parameters.

### B cell analysis

The DEGs between the indolent group and the progressive group of B cells were calculated by FindMarkers in the Seurat package; then, the DEGs were sorted with avg_log2 FC. The functional enrichment analysis was performed using the GSEA algorithm [48] in the "clusterProfile," which supports statistical analysis and visualization of functional profiles for genes and gene clusters. In GSEA, 50 hallmark gene sets in MSigDB were used for annotation. The software was run with default parameters.

### Cell–cell interaction analysis

CellPhoneDB (https://www.cellphonedb.org), a Python-based tool for systematically analyzing cell–cell communication networks [49], was used to infer ligand-receptor interactions among all cell types. Ligand-receptor interactions between two cell clusters were identified based on the specific expression of a receptor by one cell population and a ligand by another cell type.

### Analysis of bulk RNA-seq data

We utilized bulk RNAseq data for PTC patients, which is available in the TCGA database. Given the unique immune microenvironment of autoimmune thyroid disease, the analysis was limited to cases without autoimmune thyroid disease (Hashimoto's and Grave's disease). Briefly, we derived gene sets that were reflective of the cell populations of interest and determined an enrichment score for each patient in the TCGA database; the Xcell score was used to evaluate the score of B cell gene sets between different T stages of PTC patients. The individual gene analyses were performed using the Stat_compare_means function in the "ggpubr" package.

### Cell lines

Human PTC cell lines K1 and B-CPAP cells were obtained from the Chinese Academy of Science cell bank. K1 cells were cultured in high (25 mM) glucose Dulbecco's modified Eagle's medium (DMEM, Gibco). B-CPAP cells were cultured in RPIM 1640 medium (Gibco). All mediums were supplemented with 10% fetal bovine serum (FBS) and 200 U/mL penicillin–streptomycin. All cell lines used in this study were authenticated by STR sequencing.

### B cell isolation and co-culture

Tumor tissue was collected from patients with PTC, and the mononuclear cells among tumors were separated using the collagenase class II. Then, CD20^+^ B cells were purified from mononuclear cells using a CD20 MicroBeads human kit (Miltenyi Biotec) according to the manufacturer's instructions. CD20^+^ B cells were cultured in RPMI 1640 Medium supplemented with 10% FBS and 200 U/mL penicillin–streptomycin. After being sorted for 24 h, CD20^+^ B cells were collected for co-culture with K1 or BCPAP. For co-culture experiments, CD20^+^ B cells with K1/BCPAP at a ratio of 1:1 and 1:2 through a transwell chamber. Another group, K1/BCPAP, was treated with a supernatant of CD20^+^ B cells. Cultured for two days, the K1/BCPAP cells were collected for CCK8 assays. The OD450 absorbance in K1/BCPAP cells was analyzed.

### Trans-well migration assays

Migration assay was performed using the Costar Transwell Invasion chamber. Transwell inserts were hydrated with serum-free DMEM for 30 min. Peripheral blood (1 × 10^4^) cells from the same healthy volunteer were obtained. CD20^+^ B cells were subsequently isolated and resuspended in serum-free RPIM 1640 medium. Thyroid cells from the indolent or progressive group were added to the lower wells of the 24-well plate, then the CD20^+^ B cells were added to the upper chamber. The medium was discarded after 24 h. Nonmigratory cells were removed with cotton-tipped swabs, and the lower surface of the insert was stained with 0.5% crystal fast violet. The cells were counted and captured under a Nikon Eclipse 80i microscope at 10 × magnification.

### Cell cycle by flow cytometry

CD20^+^ B cells of tumor tissue from patients with PTC were obtained according to the manufacturer's instructions. The CD20^+^ B cell cycle was monitored by flow cytometry with PI/RNase staining buffer (BD Biosciences). Briefly, the cells were harvested, fixed with 75% ethanol at −20 °C overnight, and stained with PI/RNase staining buffer for 15 min. The cell cycle analyses were performed with a NovoCyte instrument (ACEA Biosciences), and the data were analyzed using NovoExpress software (ACEA).

### Multiplex immunohistochemical staining

Multiplex immunohistochemical staining of the tissue was performed using a multiple fluorescent IHC staining kitTG (TissueGnostics). Briefly, the tissue was baked at 60 °C for 1 h and then deparaffinized, and the antigen was retrieved. Then, commercial hydrogen peroxidase was used to remove the endogenous peroxidase for 10 min. Next, the tissue was blocked for 20 min and incubated with one of the following antibodies to process immunofluorescence staining for 1 h: anti-CD4 (1:500, Abcam), anti-CD8 (1:1000, Abcam), anti-CD20 (1:100, Abcam), and anti-CD21 (1:8000, Abcam). After washing the Tissue with PBS, we incubated it with the secondary antibody (Abcam) for 20 min. Next, TG dyes (TG480/520/570/620/700, TG) were added and incubated at room temperature for 10 min. Microwave treatment was used to remove the antibody complex. Subsequent markers were counterstained, and the steps were repeated until all indicators were assessed. Slides were counterstained with DAPI (Beyotime) for 5 min and were enclosed with an anti-fade mounting medium. Finally, TissueFAXS (TissueGnostics) with a Zeiss Axio Imager Z2 Microscope System was applied to acquire the images, and StrataQuest software (TissueGnostics) was used to quantify the cell density of nucleus area per cell, expression per cell, and area per cell.

### Statistical analysis

The two-sided unpaired Wilcoxon test was performed in Prism 10 to compare the two independent groups. The test ranks all values from both groups and calculates the test statistic based on the sum of ranks, with the null hypothesis stating no difference between the two groups. The p-value was then calculated to determine the significance of the difference. The results were visualized with Prism 10, providing test statistics, p-value, and a graphical representation of the ranked distributions. Statistical significance was assessed with a threshold of *p* < 0.05.

## Supplementary Information

Supplementary Material 1

## Data Availability

The raw sequence data reported in this paper have been deposited in the Genome Sequence Archive in National Genomics Data Center [50], China National Center for Bioinformation / Beijing Institute of Genomics, Chinese Academy of Sciences (GSA-Human: HRA009354) that are publicly accessible at https://ngdc.cncb.ac.cn/gsa-human.
