# Supplementary material for: Single-cell transcriptomics analysis reveals that the tumor-infiltrating B cells determine the indolent fate of papillary thyroid carcinoma
Source: J Exp Clin Cancer Res. 2025 Mar 11;44:91. doi: 10.1186/s13046-025-03341-7 (PMC11895268; doi:10.1186/s13046-025-03341-7)
Supplement: Supplementary file 1 — Supplementary Material 1 [file 13046_2025_3341_MOESM1_ESM.docx]

Supplementary Materials for

**Single-cell transcriptomics analysis reveals that the tumor-infiltrating B cells determine the indolent fate of papillary thyroid carcinoma**

Chunmei Li *et al.*

*Corresponding author. Email: licm89@126.com

**This PDF file includes:**

Figs. S1 to S18

**Supplementary Figures:**


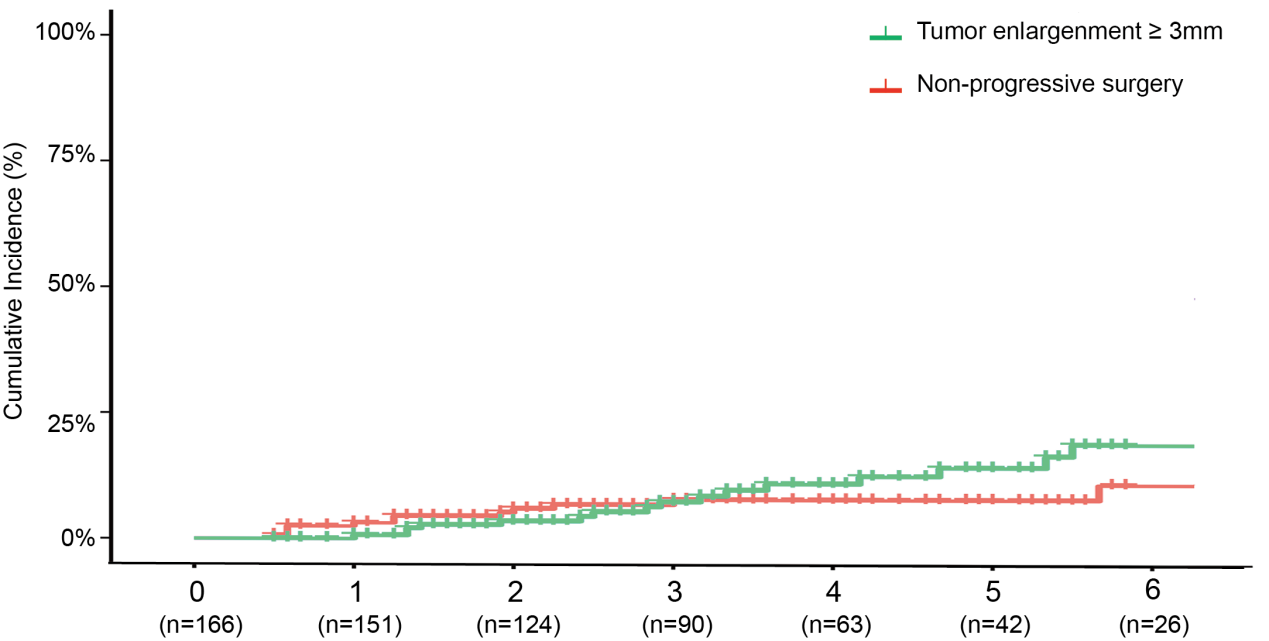


**Supplementary Fig. 1: Kaplan–Meier curves for the cumulative incidence of PTCs.** The six-year cumulative incidence of outcomes in the AS cohort of patients with low-risk PTC. AS, active surveillance.


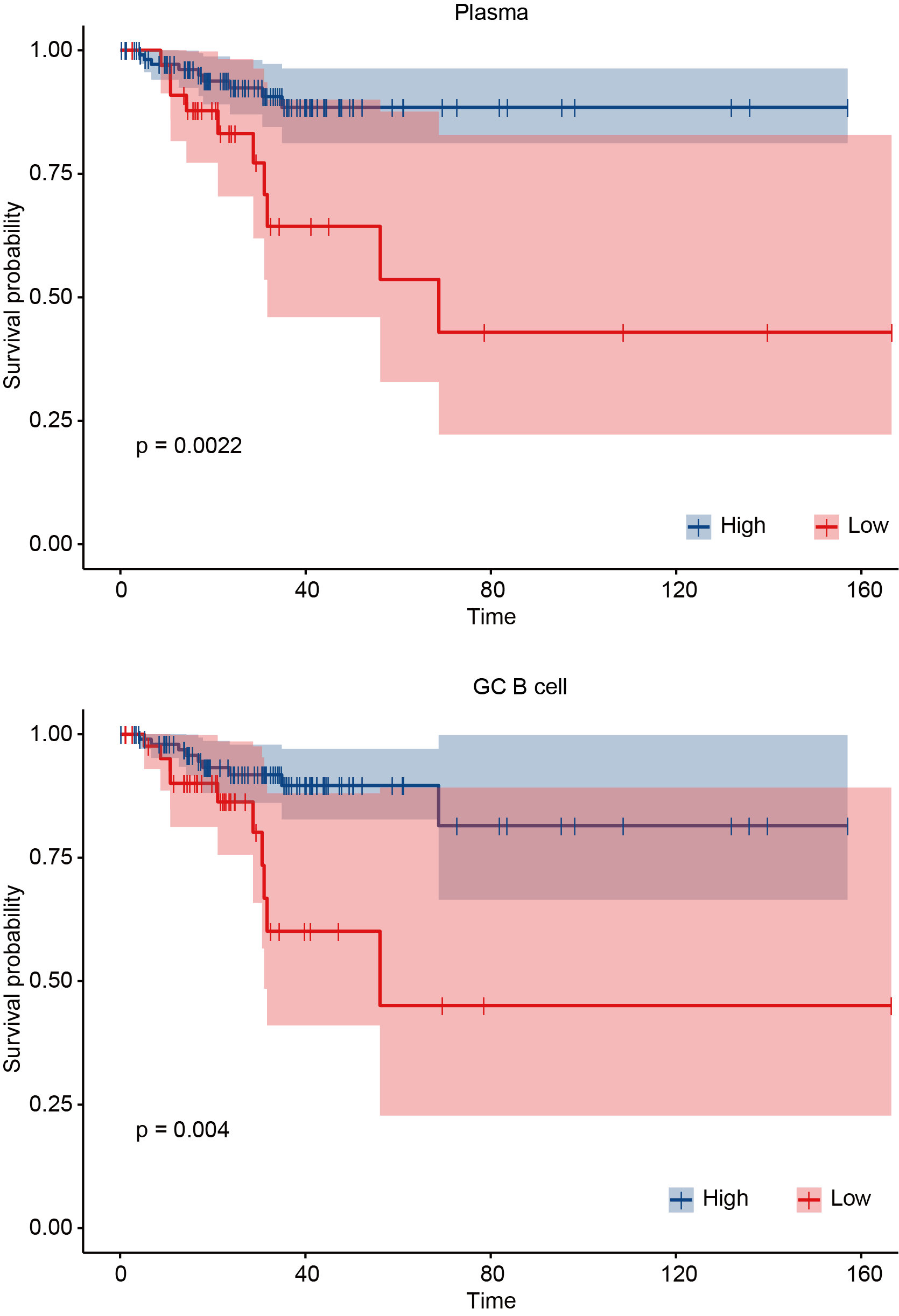


**Supplementary Fig. 2:** **Kaplan–Meier plot of DFS for PTC patients in the TCGA database.** The DFS analysis was based on enrichment scores for plasma (up) and GC-B (down) cells (Methods). Cox proportional hazard models using a log-rank test were used for DFS analysis. DFS, Disease-Free Survival.


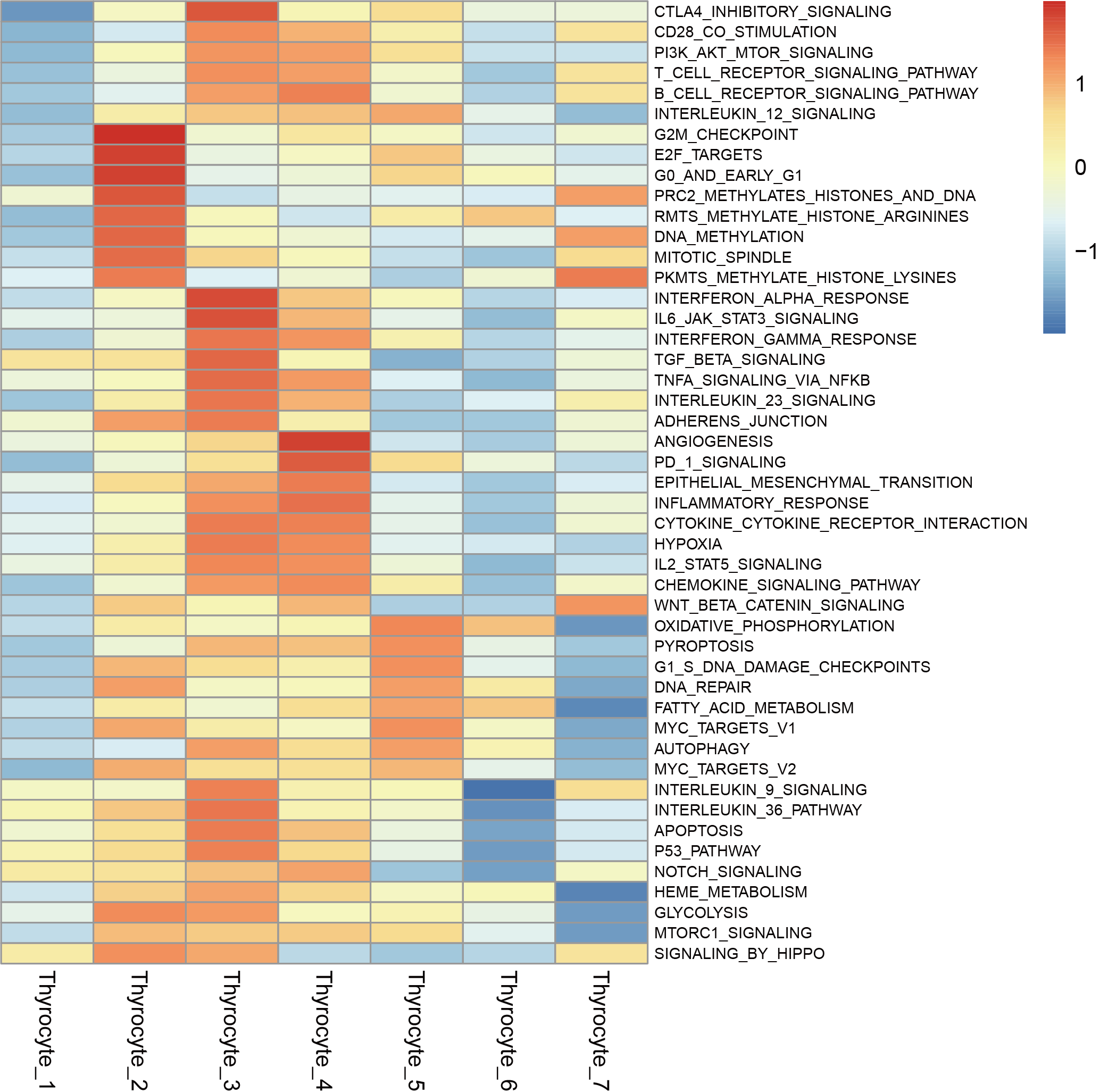


**Supplementary Fig. 3:** **Heatmap of the GSVA pathway enrichment scores of each thyrocyte cluster.** GSEA of DEGs using hallmark gene set collections. The most enriched hallmarks (ranked by the FDR q-values) are shown with the number of mapped and total genes in the pathways. Pearson correlation analysis was performed based on the mean expression levels of the genes involved in these hallmarks. DEGs, Differentially Expressed Genes.


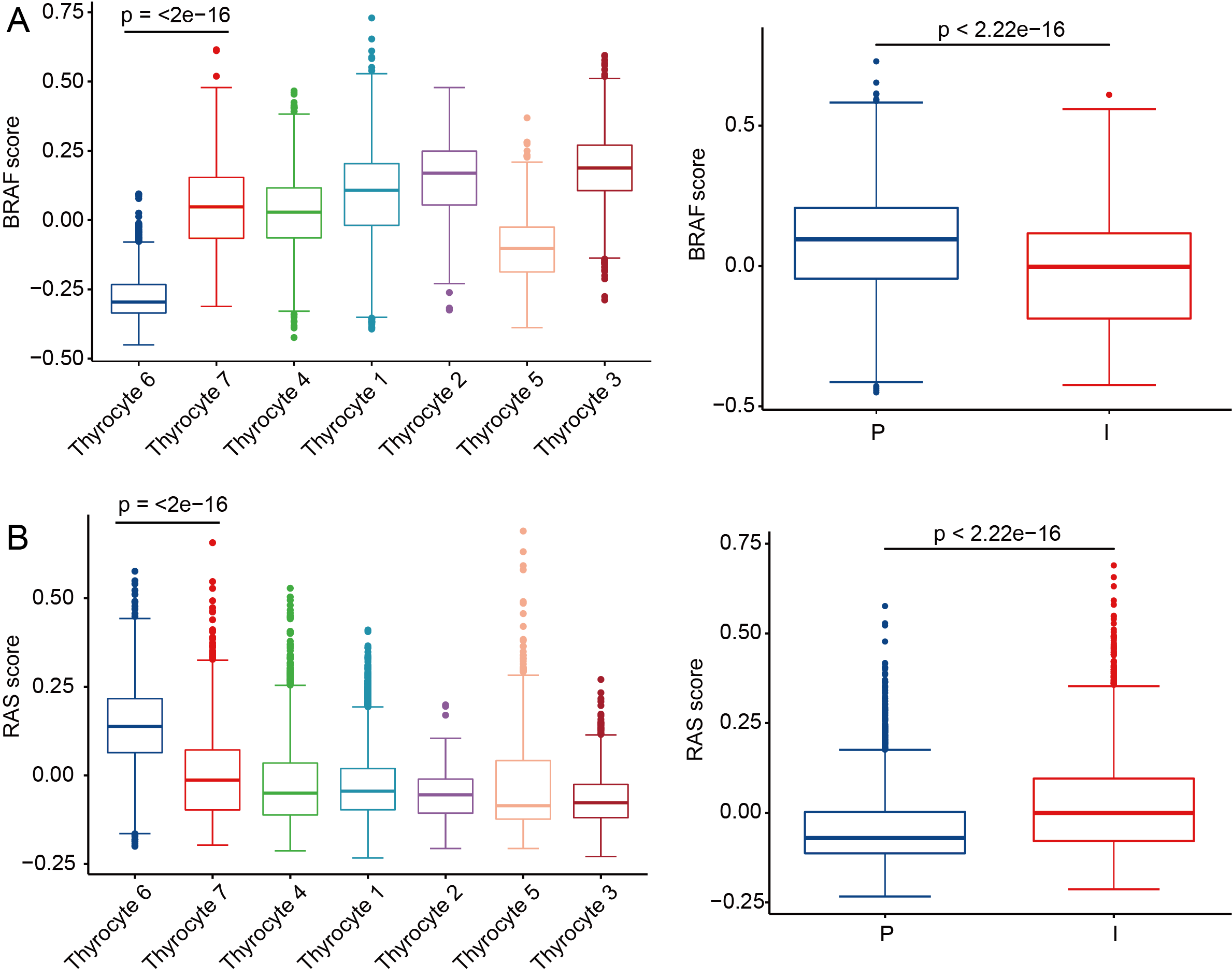


**Supplementary Fig. 4: The mutation profiles of thyrocytes.** (A) Boxplots of the BRAF^V600E^ scores of each thyrocyte cluster (left) and tissue type (right). (B) Boxplots of RAS scores of each thyrocyte cluster (left) and tissue type (right). A two-sided unpaired Wilcoxon test was performed to compare between groups.


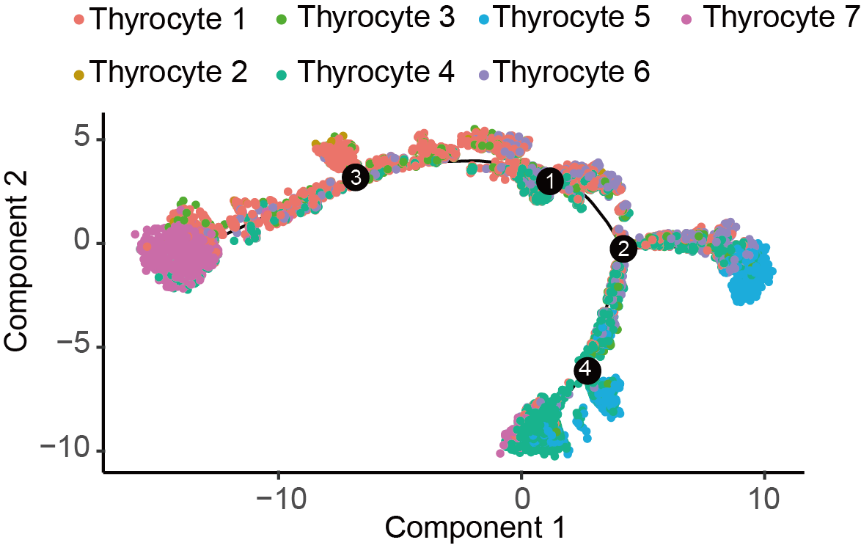


**Supplementary Fig. 5:** **The pseudo-time trajectories of all thyocytes.** Thyrocytes were ordered along pseudotime trajectories, with the cells color-coded by cell cluster.

**
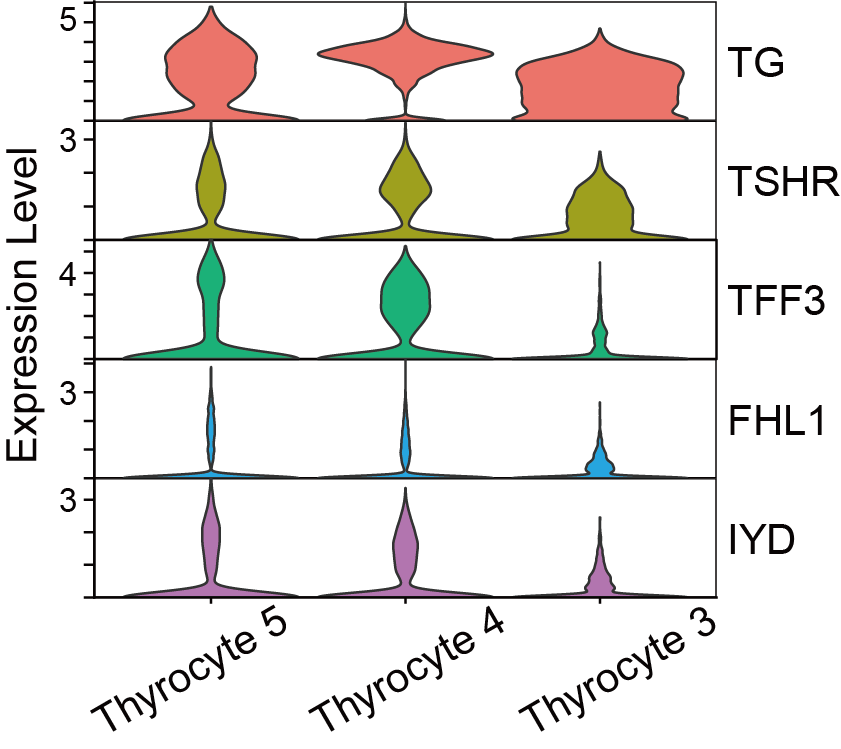
**

**Supplementary Fig. 6: Violin plots of several DEGs between thyocyte 5, 4, and 3 clusters.** The expression level of *TG*, *TSHR*, *TFF3*, *FHL1,* and *IYD* decreases continuously from thyrocyte 5 and thyrocyte 4 to thyrocyte 3. DEGs, Differentially Expressed Genes.


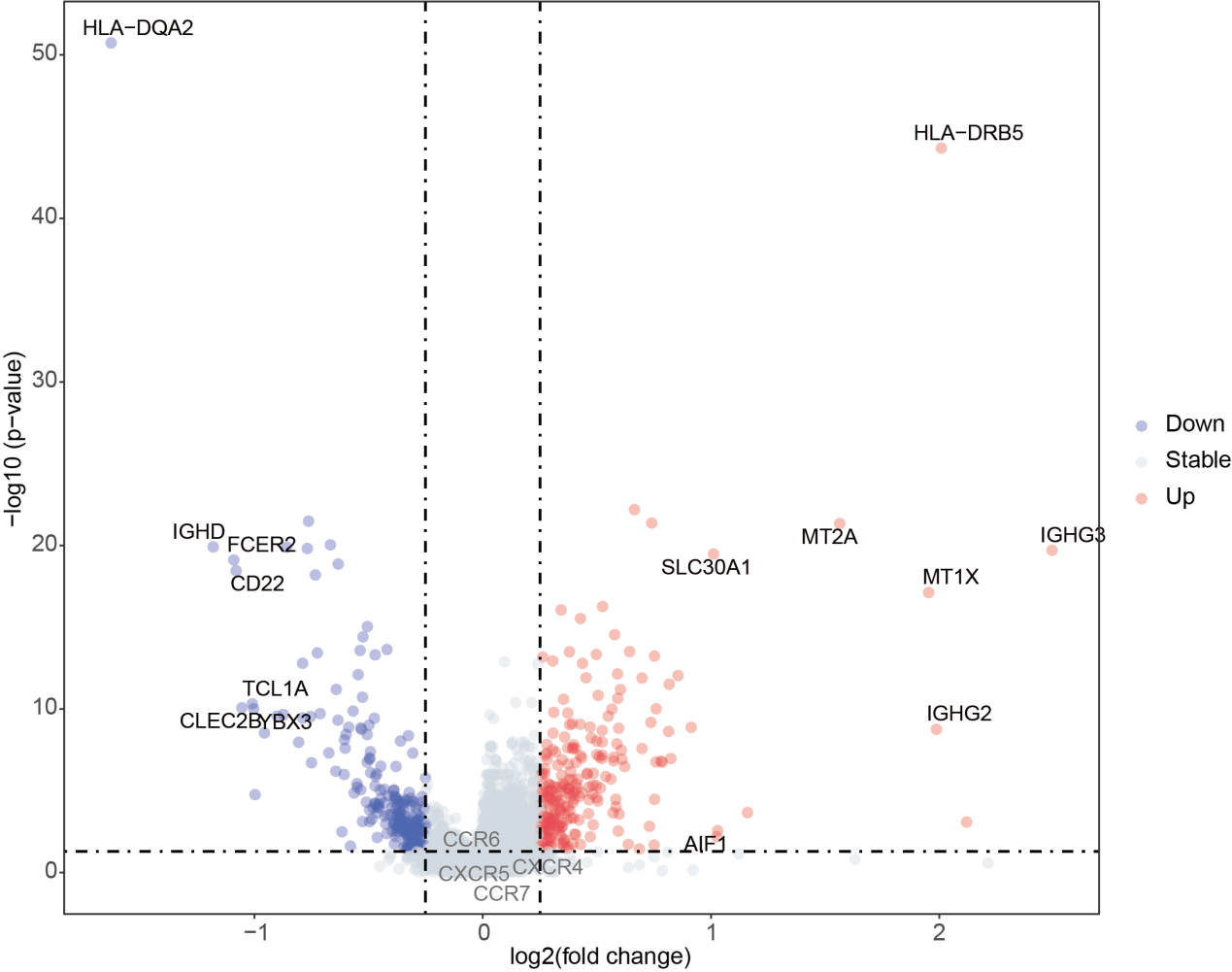


**Supplementary Fig. 7: Volcano plot of the differential expression of B lymphocyte recruitment-related receptors genes between the indolent and progressive groups.** Red and blue dots represented the genes upregulated and down-regulated, respectively.


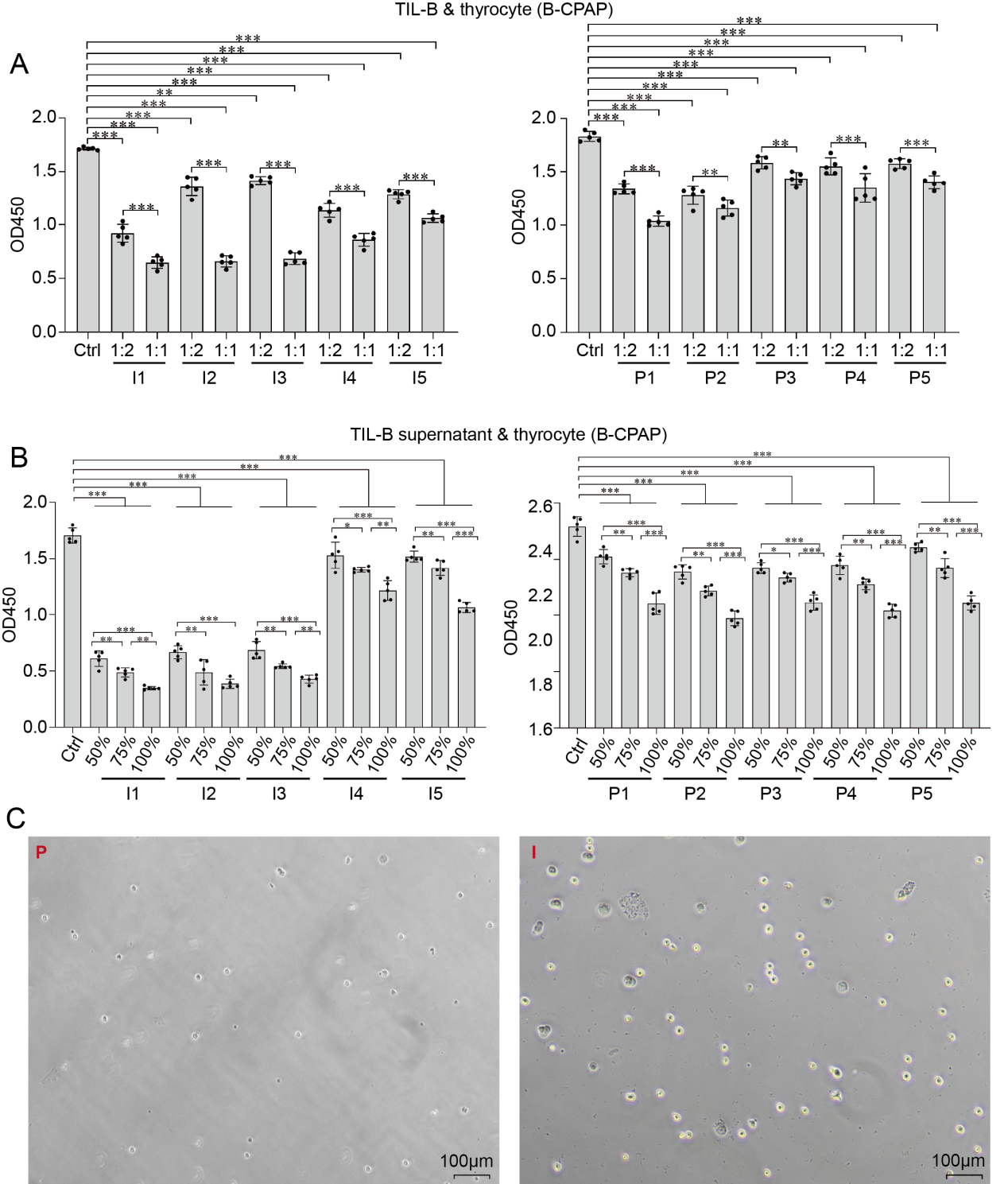


**Supplementary Fig. 8: Cell growth of BCPAP cells was inhibited by TIL-B cells.** Cell growth of BCPAP cells was inhibited by TIL-B cells (A) and culture supernatants (B), both from indolent and progressive PTCs. Cell growth was determined by using CCK8 assays. (C) The snapshot of peripheral B cell recruitment results by thyroid cells from the progressive and indolent PTCs. A two-sided unpaired Wilcoxon test was performed to compare between groups. * indicates p-value < 0.05, ** indicates p-value < 0.01, *** indicates p-value < 0.001.


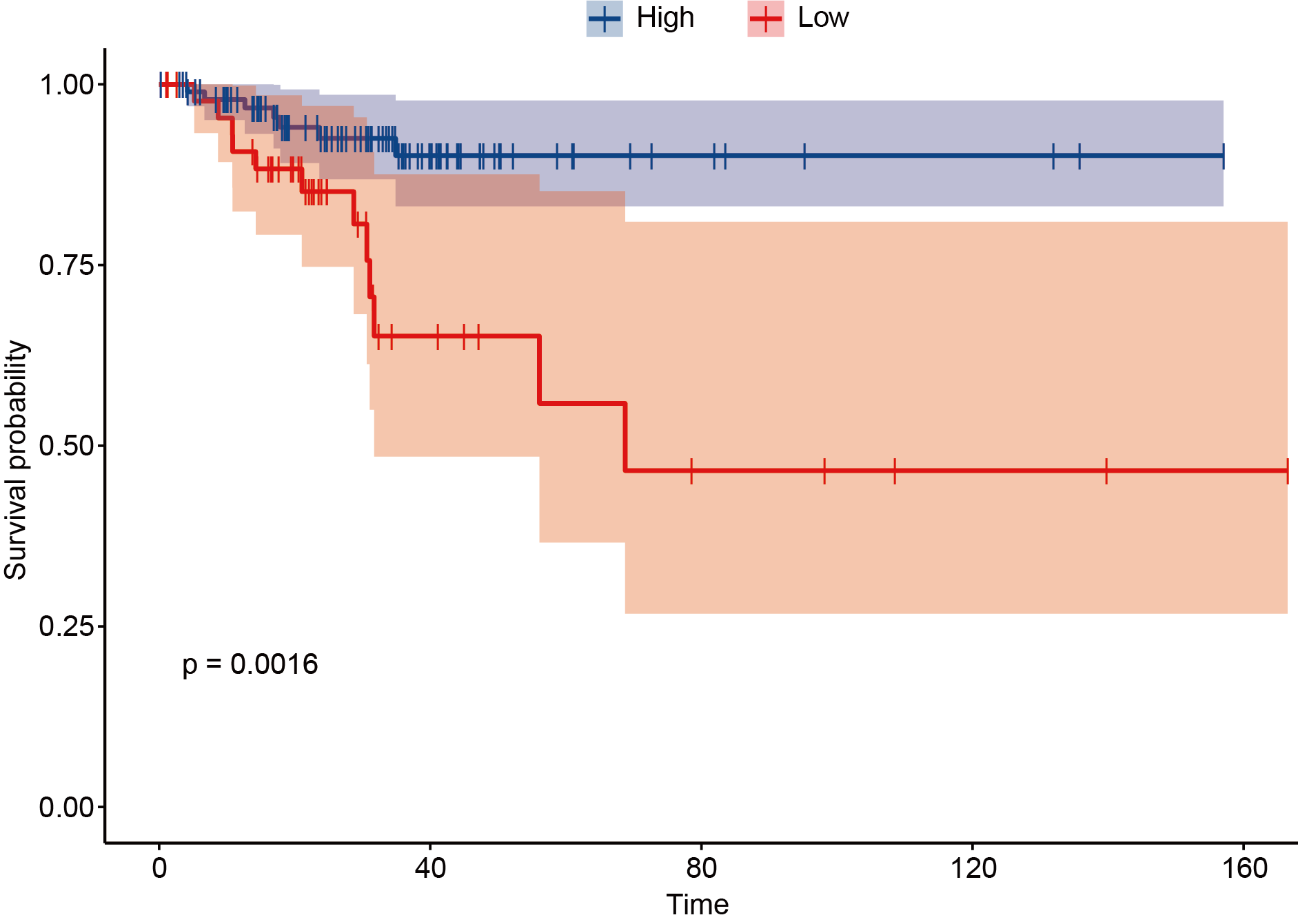


**Supplementary Fig. 9: Kaplan–Meier plot of DFS for PTC patients in the TCGA database.** DFS analysis was based on enrichment scores for tertiary lymphatic structure using the Xcell method. Cox proportional hazard models with a log-rank test were used for the DFS analysis.


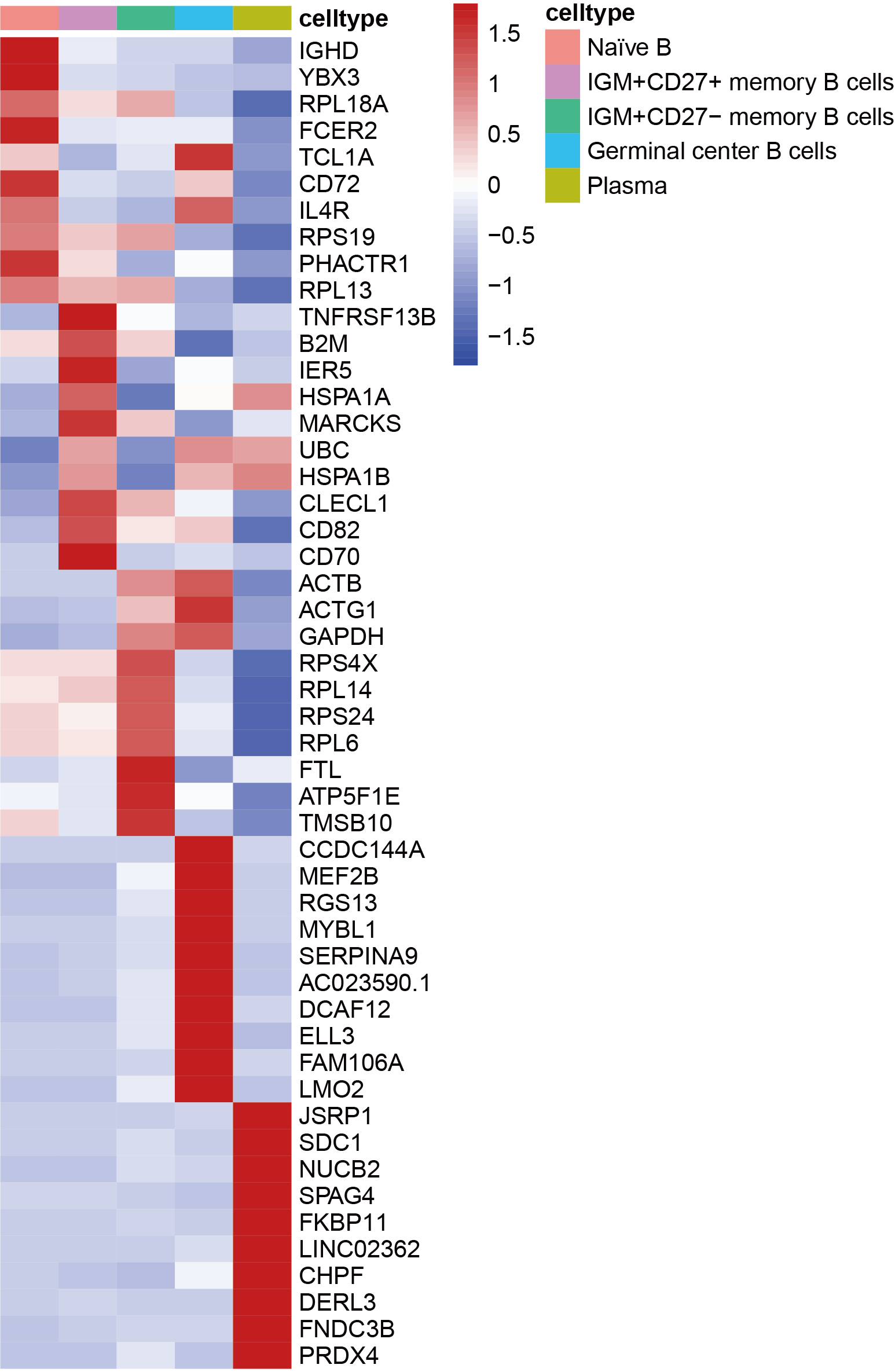


**Supplementary Fig. 10: Heatmap of genes enriched for expression in each B cell cluster.** Cell clusters are indicated on top. Red indicated upregulated genes, and blue indicated down-regulated genes.


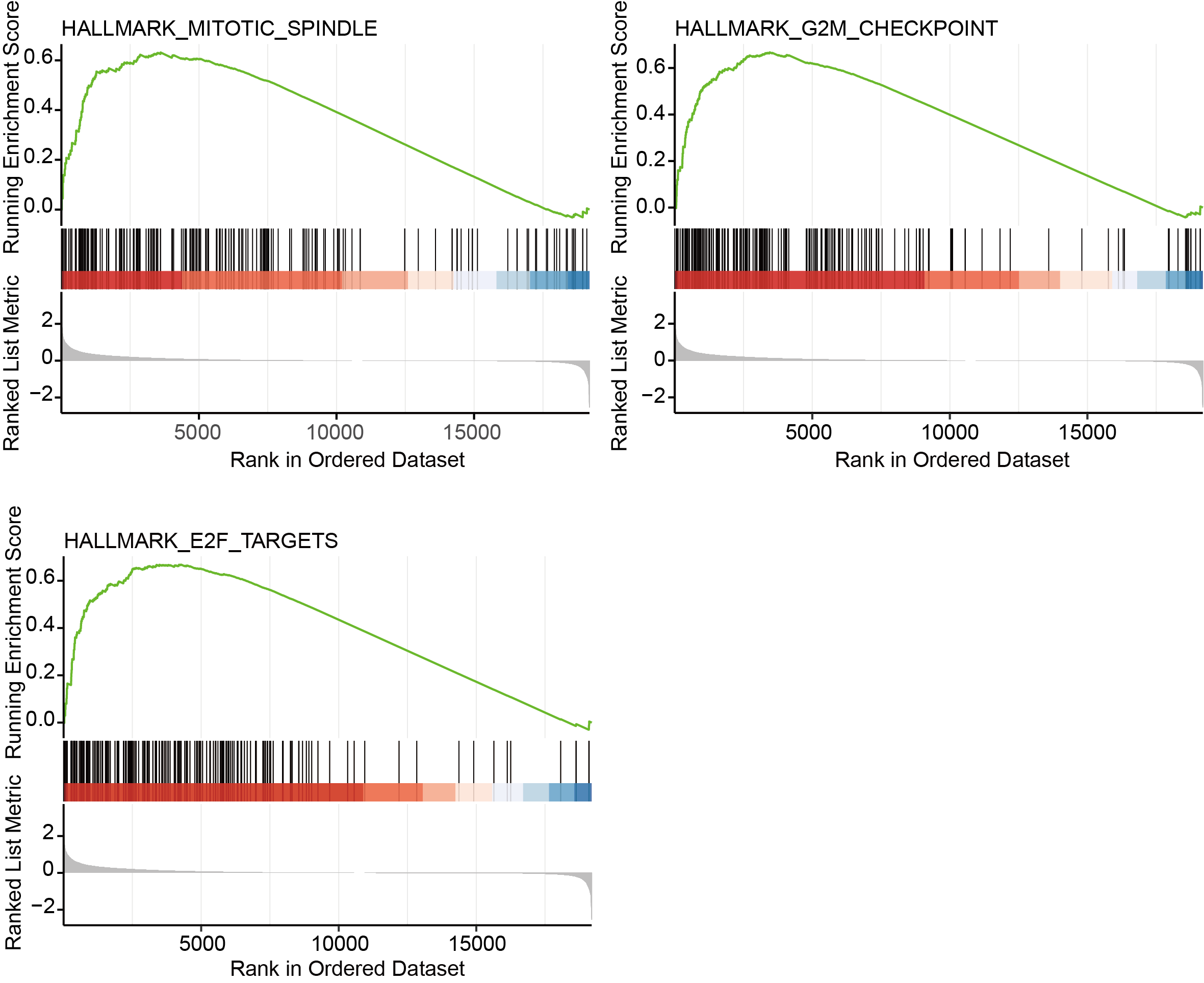


**Supplementary Fig. 11: Gene set enrichment analysis (GSEA) results of the enrichment of three cell cycle and mitotic spindle-related pathways in GC-B cell.** Gene sets of “E2F_TARGETS”, “G2M_CHECKPOINT”, and “MITOTIC_SPINDLE” were significantly upregulated in GC-B cells.GSEA analysis using hallmark gene sets from MsigDB.


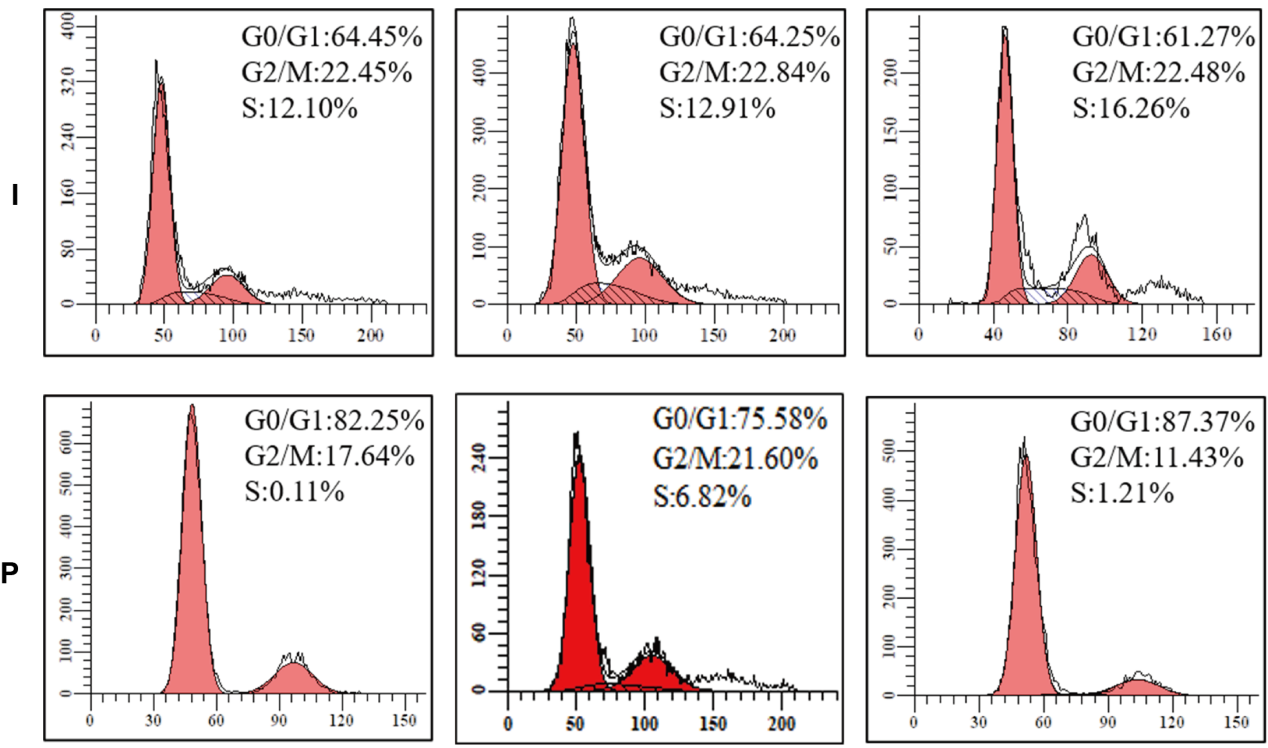


**Supplementary Fig. 12: Flow cytometry analysis of the cell cycle profiles of TIL-B cells of the indolent and progressive groups.** Cells were fixed with 75% ethanol and stained with PI/RNase.


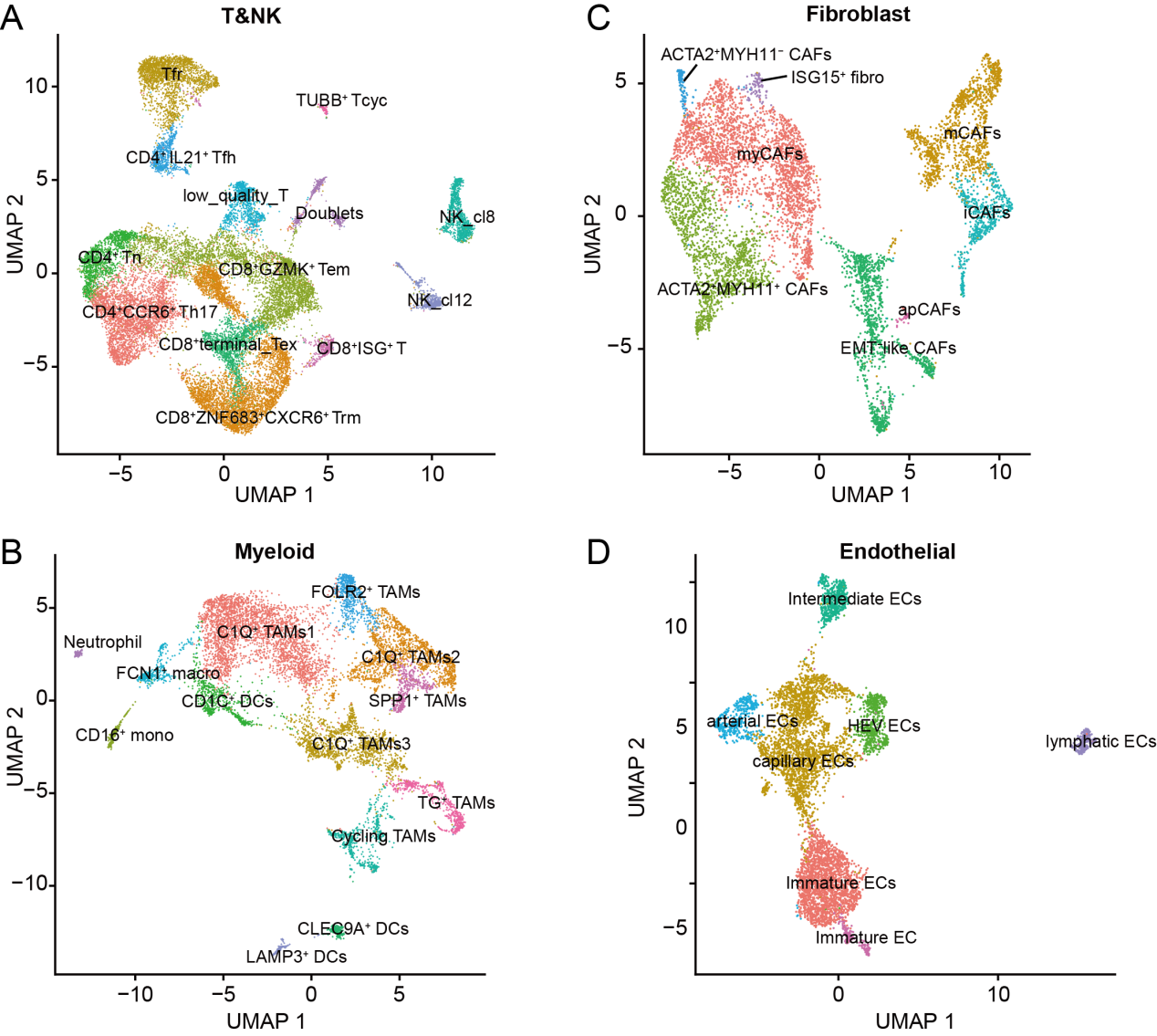


**Supplementary Fig. 13:** **UMAP plot of the T&NK (A), myeloid (B), fibroblast (C), and endothelial (D) from early-stage PTCs.** Each dot represents one cell, color-coded by cell type.


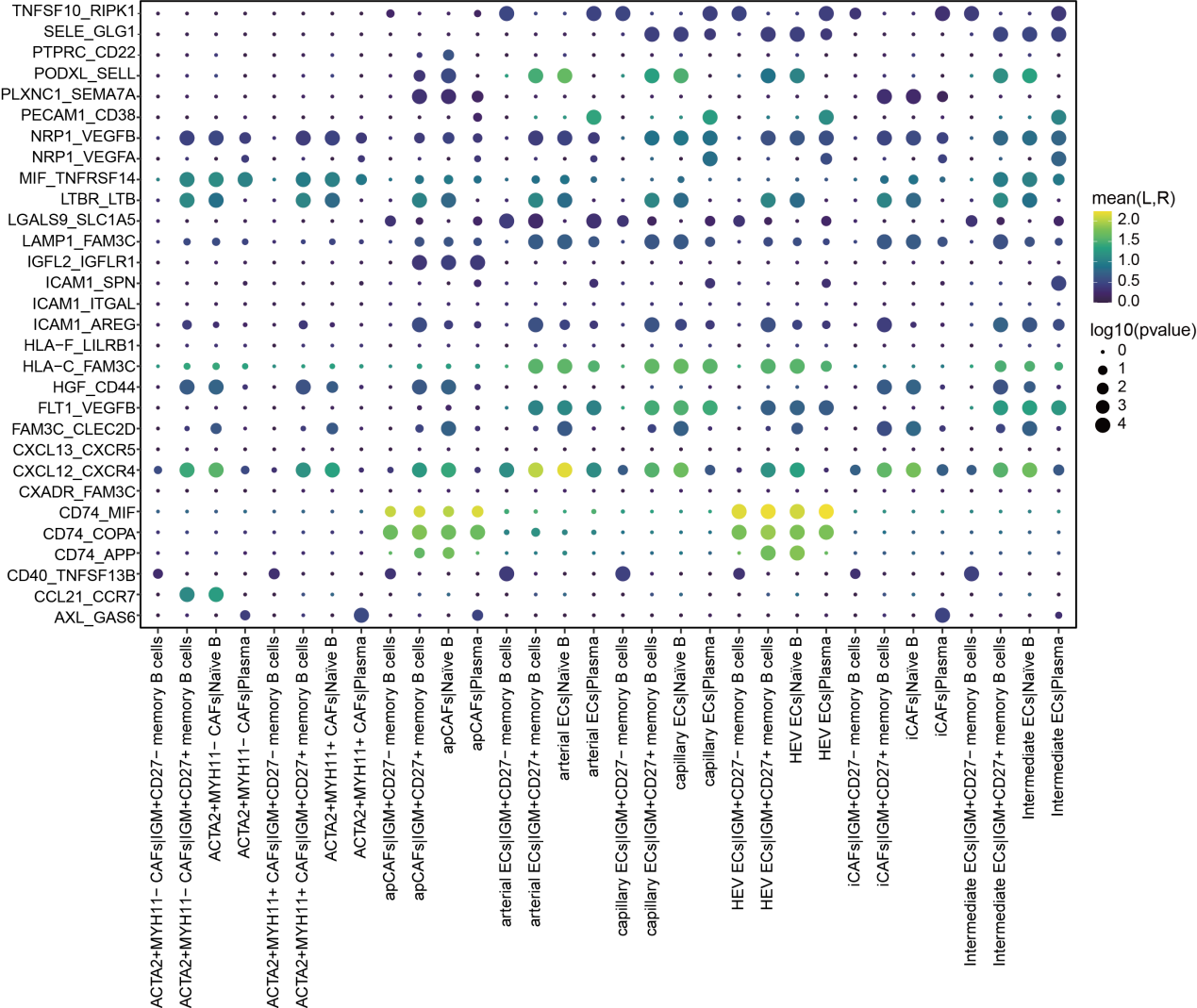


**Supplementary Fig. 14:** **Bubble plots of** **cell-cell communication between T/DC cells and B cells in the indolent group.** The bubble color presented the interaction intensity, and the bubble size presented the averaged scaled interaction value. CellphoneDB established the interaction network.


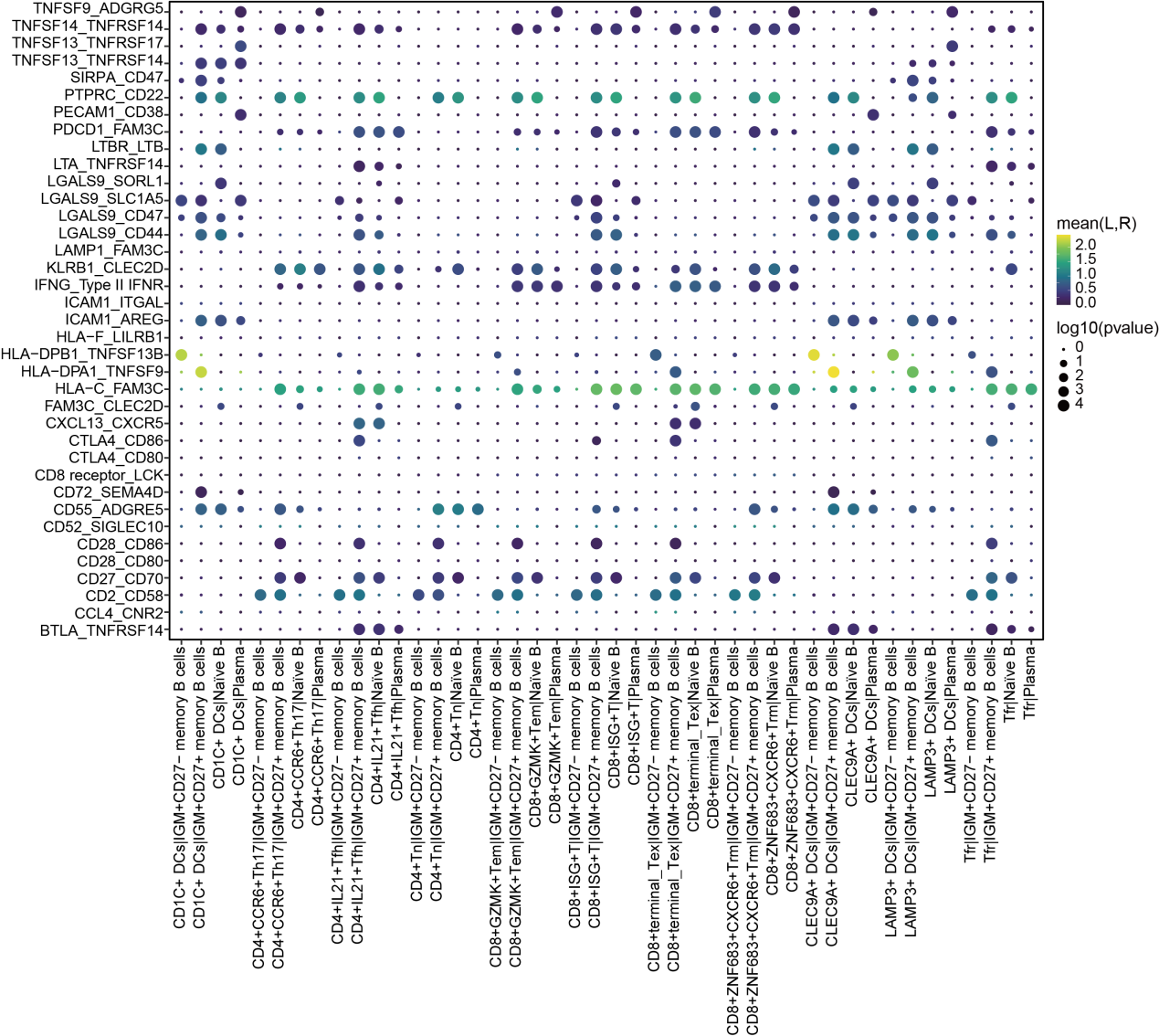


**Supplementary Fig. 15:** **Bubble plots of cell-cell communication between T/DC cells and B cells in the progressive group.** The bubble color presented the interaction intensity, and the bubble size presented the averaged scaled interaction value. CellphoneDB established the interaction network.


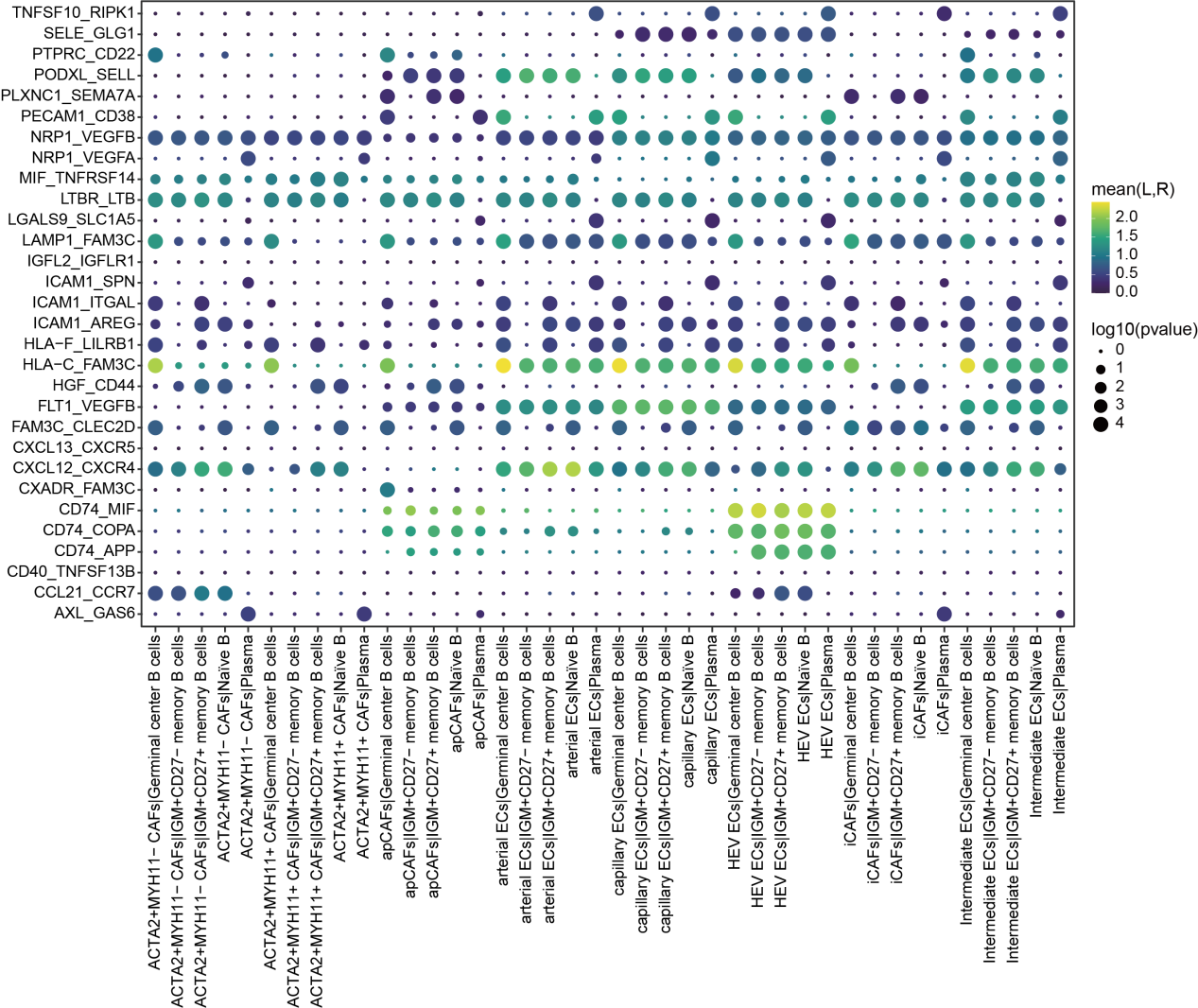


**Supplementary Fig. 16: Bubble plots of cell-cell communication between fibroblasts/ endothelial cells and B cells in the indolent group.** The bubble color presented the interaction intensity, and the bubble size presented the averaged scaled interaction value. CellphoneDB established the interaction network.


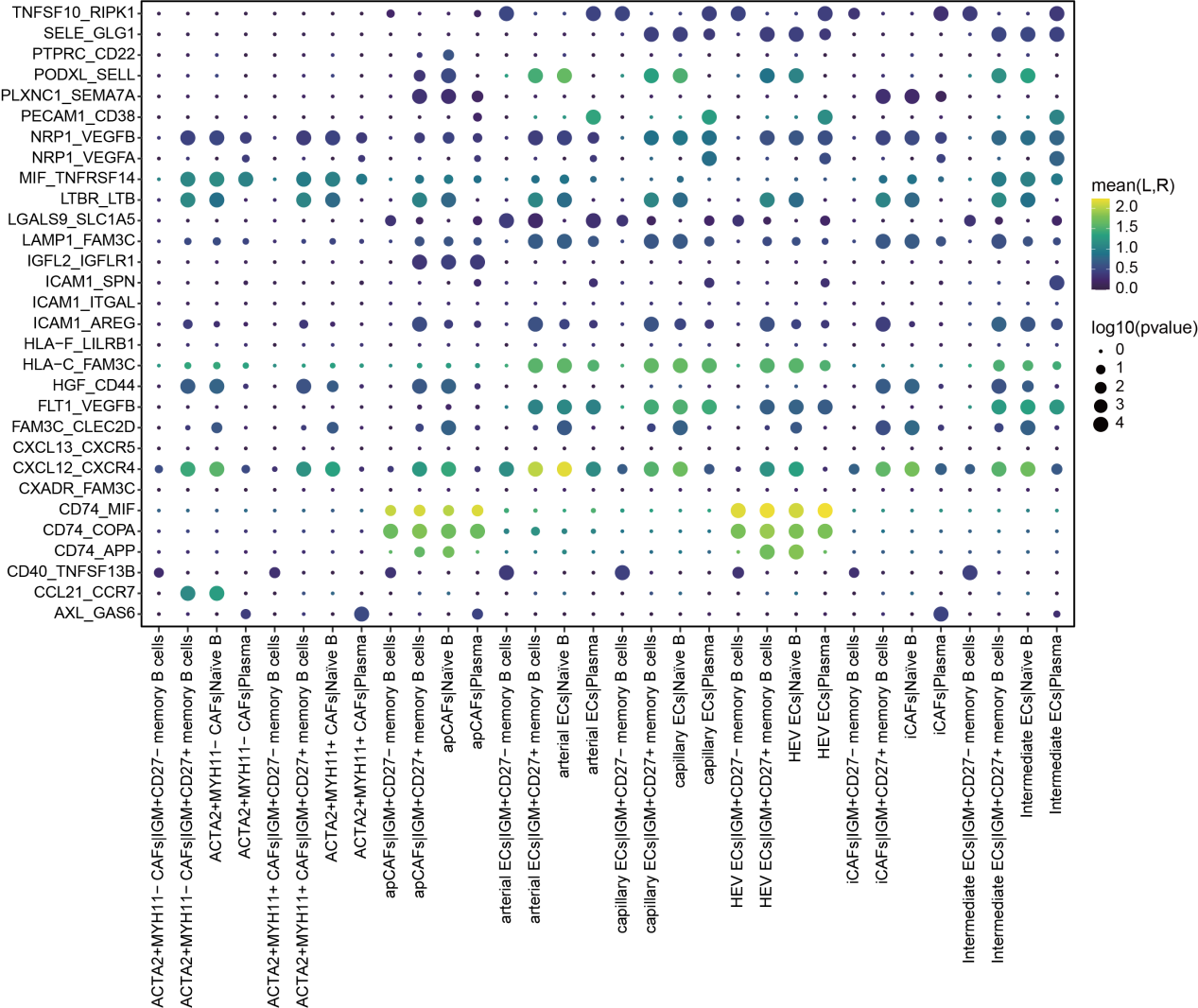


**Supplementary Fig. 17: Bubble plots of cell-cell communication between fibroblasts/ endothelial cells and B cells in the progressive group.** The bubble color presented the interaction intensity, and the bubble size presented the averaged scaled interaction value. CellphoneDB established the interaction network.


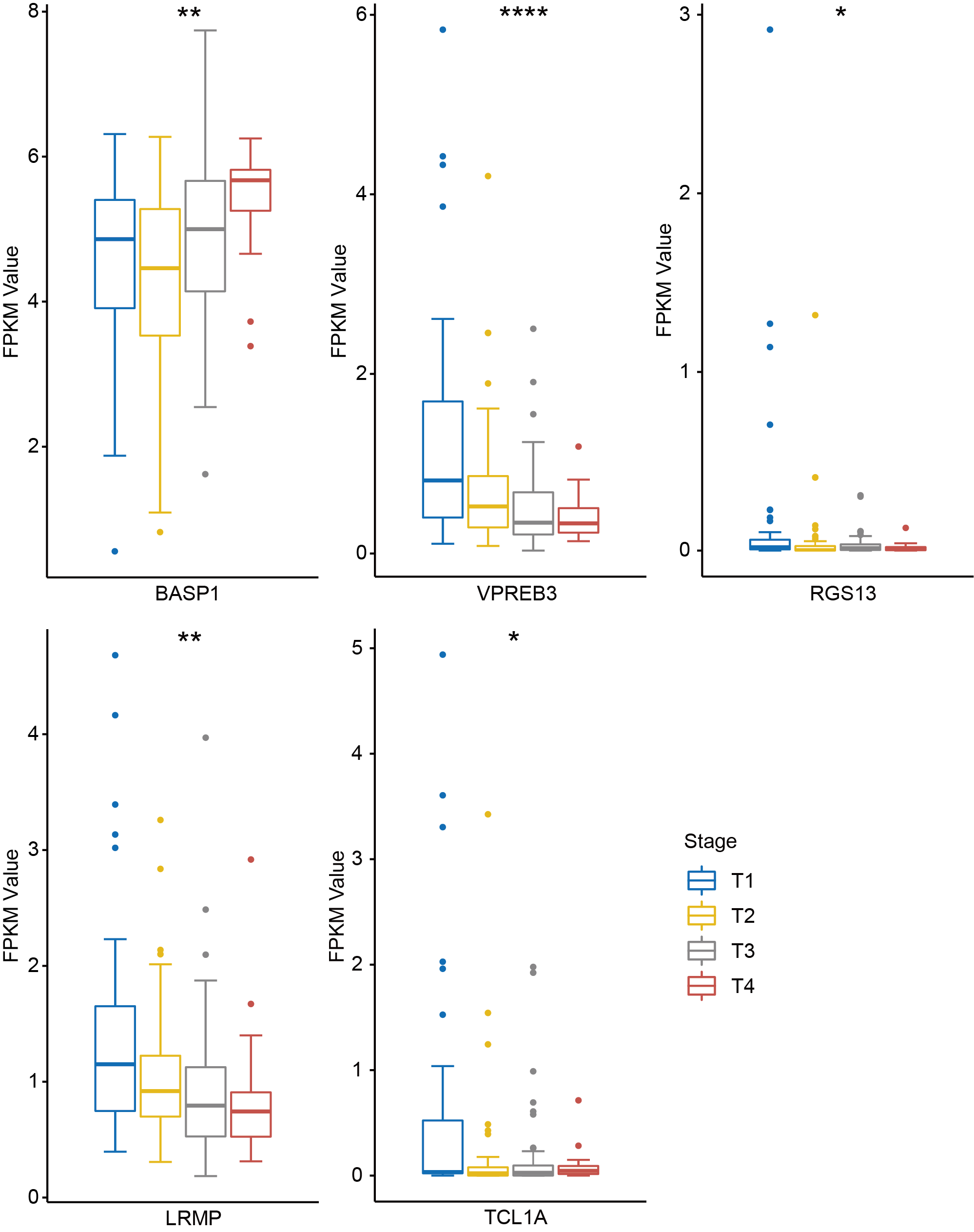


**Supplementary Fig. 18:** **Boxplot of the GC-B-specific gene expression i****n different T-stages of PTCs.** The PTC data from the TCGA database. The results showed that GC-B cells are positively associated with improved T stages for the PTC patients. A two-sided unpaired Wilcoxon test was performed to compare between groups. * indicates p-value < 0.05, ** indicates p-value < 0.01, *** indicates p-value < 0.001.
